# Genome-wide investigation of ABCB, PIN, and AUX/LAX gene families and their involvement in the formation of leaf protrusions in *Sesamum indicum*

**DOI:** 10.3389/fpls.2024.1526321

**Published:** 2025-01-31

**Authors:** Yanli Li, Yujia Ma, Huihui Gong, Xinxiao Cui, Xin Wang, Yuling Dong, Ying Chen, Junsheng Zhao

**Affiliations:** ^1^ Institute of Industrial Crops, Shandong Academy of Agricultural Sciences, Jinan, Shandong, China; ^2^ College of Life Sciences, Shandong Normal University, Jinan, Shandong, China; ^3^ Crop Research Institute, Shandong Academy of Agricultural Sciences, Jinan, Shandong, China

**Keywords:** PAT, ABCB, PIN, LAX, *Sesamum indicum*, leaf protrusion

## Abstract

*Sesamum indicum*, a highly esteemed oil crop, has exhibited remarkable value and potential in diverse areas encompassing the economy, food industry, and health. We have observed that there are small protrusions on the leaves of the indehiscent capsule material G1358. No obvious difference was detected on overall auxin content between the leaves of G1358 and LZ1 from metabolomic analysis. However, auxin levels at the base of G1358 leaves were notably higher than in LZ1, suggesting a correlation between the small protrusions at the base and polar auxin transport (PAT). PAT is essential for regulating growth and development across different plant tissues. PAT primarily relies on three families of transporter proteins: ABCB, PIN, and AUX/LAX. However, the ABCB, PIN, and AUX/LAX protein families in *Sesamum indicum* have not been systematically characterized. Herein, we identified 21 SiABCBs, 11 SiPINs, and 5 SiLAXs in *S. indicum*. Our analysis indicated that tandem duplications have facilitated the expansion of SiLAX, SiPIN, and SiABCB gene families, which have undergone purifying selection throughout their evolutionary history. Transcriptome screening and RT-qPCR analysis revealed that *SiABCB3*, *SiABCB6*, and *SiPIN10* positively regulate PAT, whereas *SiABCB7* and *SiABCB9* negatively regulate PAT in G1358. These regulatory interactions contribute to the formation of small protrusions in G1358 leaves and enhance the rate of photosynthesis. Our findings provide a theoretical foundation for understanding PAT genes and their roles in the environmental adaptation of sesame.

## 1 Introduction

*Sesamum indicum*, a crucial oilseed crop, is valued not only for its nutritional benefits but also for its applications in traditional medicine due to its potential therapeutic properties. However, its cultivation is limited by low yields, necessitating efforts to select varieties with high quality and enhanced photosynthetic efficiency (Pathak et al., 2017; Wei et al., 2022).

Auxin, a crucial plant hormone, is responsible for diverse physiological processes such as organogenesis, morphogenesis, gravitropism, apical dominance, and tissue differentiation (Goldsmith, 1993; Bohn-Courseau, 2010; Zhao, 2010). The process of polar auxin transport (PAT) is particularly important, as it involves the directional movement of auxin from the shoot tip toward the base of the stem. This directional transport is crucial for maintaining growth and developmental balance across different plant tissues (Jensen et al., 1998). PAT relies on three main transporter families: ABCB, PIN, and AUX/LAX proteins, which establish auxin concentration gradients essential for these developmental processes (Peret et al., 2012; Geisler et al., 2017; Zhou and Luo, 2018). However, the ABCB, PIN, and AUX/LAX gene families in *S. indicum* have yet to be characterized.

ABCB proteins, which are part of the ATP-binding cassette (ABC) transporter superfamily, play a significant role in mediating PAT. Evidence suggests that ABCB19, a member of this family, influences auxin distribution within plants, affecting processes such as hypocotyl growth during seedling establishment (Wu et al., 2010), phototropic and gravitropic responses (Nagashima et al., 2008), hypocotyl elongation (Wu et al., 2016), cotyledon development (Lewis et al., 2009), and anisotropic cell expansion (Zhang et al., 2024a). ABCB proteins are typically located uniformly localized at the plasma membrane and are crucial for the directional movement of auxin through tissues (Cho and Cho, 2013). The synergistic effect of ABCB4 and PIN2 on auxin transport selectivity highlights the importance of these proteins in PAT (Ep and Sd, 2021). PIN proteins, localized specifically at the plasma membrane, are primary drivers of PAT. They use an elevator mechanism to transport auxin anions out of the cell (Joshi and Napier, 2023). Mutations in the *PIN-FORMED* (*PIN1*) gene resulted in reduced PAT in *Arabidopsis thaliana* inflorescence axes, indicating the critical role of PIN proteins during this process (Galweiler et al., 1998). This interaction between PIN and ABCB proteins is crucial for efficient auxin transport. Previous research has shown that co-expression of PIN2 and ABCB4 markedly enhances the selectivity for IAA, suggesting a synergistic effect necessary for effective PAT (Ep and Sd, 2021). While ABCBs can mediate auxin efflux independently of PINs, PIN-regulated auxin efflux occurs predominantly in conjunction with ABCBs (Mellor et al., 2022).

Evidence suggests that the development of leaf veins, which are critical for water and nutrient transport within leaves, is closely related to PAT. This indicates that auxin’s role in leaf development extends beyond its general functions in plant growth and development. Additionally, PAT appears to influence early leaf flattening (Wang et al., 2022), indicating its importance not only for overall plant architecture but also for specific leaf morphological features. Leaf morphology, including shape, size, venation patterns, and chloroplast arrangement within leaves, significantly affects how light is absorbed, distributed, and utilized for photosynthesis. Broad or broadly lobed leaves may be more effective in capturing light due to their larger surface area and potentially stronger water potential gradients across the leaf tissue, which can enhance photosynthetic efficiency (Leigh et al., 2014). This is supported by observations that broad-lobed leaves of *Lomatia tinctoria* exhibit higher and less spatially variable heterogeneous photosynthetic efficiency compared to narrow-lobed leaves under stress conditions. However, it remains unclear whether small protrusions on leaves are related to PAT or if they influence the plant’s photosynthetic rate.

In this study, we examined the phylogeny of the ABCB, PIN, and LAX families in *S*. *indicum*. We identified 21 SiABCB family members, 11 SiPIN family members, and 5 SiLAX family members, in *S. indicum*. Transcriptome and metabolome analysis revealed that, compared to *S*. *indicum* LZ1, the G1358 variety exhibited enhanced PAT mediated by ABCB and PIN proteins. This enhancement was associated with the formation of small protrusions on G1358 leaves and increased photosynthetic rates.

## 2 Materials and methods

### 2.1 Materials

The indehiscent capsule material G1358 was obtained from the Oil Crops Research Institute of the Chinese Academy of Agricultural Sciences. Luzhi No.1 (LZ1) is a broadly adaptable sesame cultivar developed by our institute.

### 2.2 Sectioning of the samples and paraffin sides

After fixing in formalin-acetic acid-alcohol (FAA) for over 24 hours, the fresh leaves were removed, and the target tissue was carefully trimmed using a scalpel under a fume hood. The trimmed tissues and their corresponding labels were transferred into an embedding frame. The tissue was then dehydrated in a dehydration box using a graded alcohol series: 75% alcohol for 4 hours, 85% alcohol for 2 hours, 90% alcohol for 2 hours, 95% alcohol for 1 hour, followed by absolute-ethanol-I for 30 minutes, absolute-ethanol-II for 30 minutes, alcohol-benzene for 5–10 minutes, xylene-II for 5–10 minutes, and melted paraffin at 65°C for three sequential stages of 1 hour each. The wax-impregnated tissue was then embedded using an embedding device. Molten wax was first poured into the embedding frame, and before it solidified, the tissues were transferred from the dehydration box to the frame, aligned as required for sectioning, and labeled. The frame was chilled on a -20°C freezing platform until the wax solidified. After removing the solidified wax block from the frame, it was trimmed and then sectioned into 4 μm slices using a paraffin microtome. The sections underwent floating on 40°C water in a spreading machine to flatten the tissue, then transferred onto glass slides and heated at 60°C in an oven. Once the wax had dried and melted, the slides were taken out and kept at ambient temperature.

### 2.3 Toluidine blue staining

The paraffin sections were sequentially immersed in dewaxing-solution-I (Servicebio) for 20 minutes, followed by dewaxing-solution-II (Servicebio) for another 20 minutes. They were subsequently treated with absolute-ethanol-I for 5 minutes, absolute-ethanol-II for 5 minutes, and rinsed with distilled water. The sections underwent staining with toluidine blue for 2 minutes, rinsing with distilled water, and microscopical examination to assess tissue coloration for appropriate differentiation. After washing with tap water, they were dried in an oven, made transparent with xylene for 5 minutes, followed by sealing with neutral gum. The sections were subsequently inspected under a microscope, and images were captured and analyzed.

### 2.4 Auxin metabolite profiling sample preparation and extraction

The processes of preparing, extracting, identifying, and quantifying samples for the targeted metabolome analysis were carried out by Wuhan Metware Biotechnology. The experiment utilized three biological replicates, with samples labeled as follows: LZ1-Leaf-1, LZ1-Leaf-2, LZ1-Leaf-3, G1358-Leaf-1, G1358-Leaf-2, and G1358-Leaf-3. Fresh leaves were collected, snap-frozen in liquid-nitrogen, crushed into powder (30 Hz, 1 minute), and kept at -80°C until further use. For extraction, plant samples (50 mg) were weighed, frozen in liquid nitrogen, and dissolved in 1 mL formic acid/water/methanol solution (1:4:15). Then, 10 μL internal standard mixture (100 ng/mL) was added to the extract for quantification. After vortexing for 10 minutes and centrifugation for 5 minutes (12,000 rpm, 4°C), the supernatants placed in a sterile plastic microtube. After evaporating the solvent to dryness, the residue was dissolved in 100 μL 80% methanol. The solution was then passed through a 0.22-μm membrane filter and prepared for LC-MS/MS assessment (Flokova et al., 2014; Li et al., 2016).

### 2.5 UPLC conditions

The samples were subjected to analysis using a UPLC-ESI-MS/MS system (UPLC: ExionLC™ AD; MS: QTRAP^®^ 6500+). The parameters included LC-column, Waters-ACQUITY-UPLC-HSS-T3-C18 (2.1 mm × 100 mm, 1.8 µm); solvent-system, water containing 0.04% acetic acid (A) and acetonitrile containing 0.04% acetic acid (B); gradient-program: initiated at 5% B (0–1 minutes), elevated to 95% B (1–8 minutes), held at 95% B (8–9 minutes), and then decreased to 5% B (9.1–12 minutes); column-temperature, 40°C; flow-rate, 0.35 mL/min; injection-volume, 2 μL (Cai et al., 2014; Xiao et al., 2018).

### 2.6 ESI-MS/MS conditions

The linear-ion-trap (LIT) and triple-quadrupole-scans were performed on a triple-quadrupole-linear-ion-trap-mass-spectrometer (QTRAP), specifically the QTRAP^®^6500+LC-MS/MS-system, equipped with an ESI-Turbo-Ion-Spray-interface. The system operated in both positive and negative ion modes and was managed using Analyst v1.6.3 software (Sciex). The ESI-source parameters included ion-source, ESI+/-; source-temperature, 550°C; ion-spray-voltage (IS), 5500 V for positive-mode and -4500 V for negative-mode; and curtain-gas (CUR) set at 35 psi. Phytohormones were assessed using scheduled multiple-reaction-monitoring (MRM). Data acquisition was carried out using Analyst v1.6.3 software (Sciex), while all metabolites underwent quantification using Multiquant v3.0.3 software (Sciex). MS parameters, such as collision-energies (CE) and declustering-potentials (DP) for each MRM transition, were further optimized. During each time period, a set of MRM transitions was monitored according to the metabolites eluting at that interval (Pan et al., 2010; Simura et al., 2018).

### 2.7 Identification of ABCB, PIN, and AUX/LAX genes in S. indicum

To identify potential *SiABCB*, *SiPIN*, and *SiLAX* genes in *S. indicum*, the auxin transporter family members from *Arabidopsis thaliana* and *Oryza sativa* were acquired (Yang et al., 2021) and BLAST searches were conducted against the *S. indicum* genome using an e-value threshold of < 1e^-10^. All candidate proteins were then validated using the NCBI CDD database (http://www.ncbi.nlm.nih.gov/cdd), InterPro (https://www.ebi.ac.uk/interpro/), and SMART (http://smart.emblheidelberg.de/). The molecular weight (MW) and theoretical-isoelectric-point (pI) of each PAT protein were predicted using the ExPASy webtool (https://www.expasy.org/) (Artimo et al., 2012). Additionally, Wolf-PSORT (https://wolfpsort.hgc.jp/) was used to predict the subcellular localizations of SiABCB, SiPIN, and SiLAX proteins (Horton et al., 2007).

### 2.8 Phylogenetic, conserved motif, gene structure, chromosomal mapping, gene duplication, and protein interaction analyses

The SiLAX, SiPIN, and SiABCB proteins were aligned using MEGA11 (http://www.megasoftware.net/). Phylogenetic trees were established using the Neighbor-Joining (NJ) method with 1,000 bootstrap replications (Tamura et al., 2021), followed by visualization with iTOL v6 (http://itol.embl.de/) (Letunic and Bork, 2021). MEME Suite (http://meme-suite.org/) was employed to identify conserved motifs within the proteins. The gene structure and conserved motif analysis were illustrated with the TBtools software v.1.098 (https://github.com/CJ-Chen/TBtools) (Chen et al., 2020). A chromosomal location map of the SiLAX, SiPIN, and SiABCB genes was created using MapChart software (Voorrips, 2002), and then enhanced for clarity with Adobe Illustrator CS6. BLAST analysis was performed to investigate the duplication patterns of PAT genes. The collinearity between chromosomes was analyzed with McscanX software (Wang et al., 2012), and the syntenic relationships of duplicated genes were illustrated using the Advanced Circos program in TBtools. Additionally, a protein-protein interaction network for SiABCB, SiPIN, and SiLAX was established using STRING (https://string-db.org/) (Szklarczyk et al., 2017) and visualized using Cytoscape.

### 2.9 Cis-acting element analysis of SiLAX, SiPIN, and SiABCB

To identify the potential cis-acting elements in *SiABCB*, *SiPIN*, and *SiLAX* gene promoter regions, 1.5 kb genomic sequences upstream of the start codon (ATG) were acquired from the PlantCARE database (http://bioinformatics.psb.ugent.be/webtools/plantcare/html/) (Lescot et al., 2002). The identified elements were visualized using the GSDS2.0 program (http://gsds.gaolab.org/) (Hu et al., 2015). Ten key cis-elements were selected for analysis, including AuxRR-core (auxin responsiveness), ARE (anaerobic induction), ABRE (abscisic acid responsiveness), CGTCA-motif (MeJA responsiveness), G-box (light responsiveness), GCN4-motif (endosperm expression), LTR (low-temperature responsiveness), Myb-binding site (CAACAG), TGA-element (auxin-responsive cis-acting element), and TCA-element (salicylic acid responsiveness).

### 2.10 RT-qPCR assays

Total RNA extraction was performed using TRIzol reagent (Tiangen Biotech, China), and cDNA synthesis was performed using a reverse-transcription-kit (R323-01, Vazyme Biotech, China). RT-qPCR assays were conducted with ChamQ-Universal-SYBR-qPCR-Master-Mix (Q711-02, Vazyme Biotech) on a LightCycler-480 instrument (Roche). The primer sequences are listed in **Supplementary Table S1**. β-Actin was used as an internal control, and relative mRNA levels were calculated using the 2^–ΔΔCt^ method (Liu et al., 2011).

### 2.11 Hormonal induction

The NAA storage solution (PH1011-100 mL, Coolaber, Beijing, China) was diluted to a concentration of 10 mg/L. At the seedling stage, NAA was uniformly sprayed onto the leaves of G1358 and LZ1, with water used as a control. Leaf specimens were harvested at 0, 6, 12, and 24 hours post-application and immediately placed in liquid nitrogen for rapid RNA extraction. RNA quality was assessed using a NanoDrop2000 ultra-microspectrophotometer (ThermoFisher), and total RNA was quantitatively analyzed by RT-qPCR.

### 2.12 RNA-seq analysis

Approximately 1.0 g of leaves from G1358 and LZ1 were rapidly frozen in liquid nitrogen and ground into a powder. Total RNA extraction was conducted using TRIzol reagent (Tiangen Biotech). Three biological replicates were conducted. Library construction and RNA sequencing were performed by Wuhan-Metware-Biotechnology (Wuhan, China). Novel transcript sequences were assembled with StringTie (Pertea et al., 2015). Annotation of these transcripts was performed by comparing them against the GO, KEGG, NR, SwissProt, trEMBL, and KOG databases. Principal-component-analysis was used to assess the correlations among replicates. Clean sequences were then aligned to the *S. indicum* reference genome (Song et al., 2023). DEGs were identified using DESeq2 with a threshold of |log_2_(Fold-Change)|≥1 and FDR<0.05 (Love et al., 2014). Gene expression patterns at various time points were analyzed by clustering genes using the K-means method.

### 2.13 Determination of chlorophyll, content photosynthetic rate and canopy apparent photosynthesis

To measure chlorophyll content, SPAD values (relative chlorophyll content) were obtained from the latest fully expanded leaf of 10 individual plants at the bud stage using a SPAD-502 Chlorophyll Meter (Konica-Minolta-Holdings, Tokyo, Japan). The photosynthetic rates (Pn) of these same leaves, as well as the canopy apparent photosynthesis for the entire population, were assessed using a Li-6800-portable-photosynthesis-system (Li-Cor, Lincoln, NE, USA). These measurements were conducted under clear, windless conditions between 9:00 and 11:00 a.m.

## 3 Results

### 3.1 Leaf morphology

**Figure 1:**
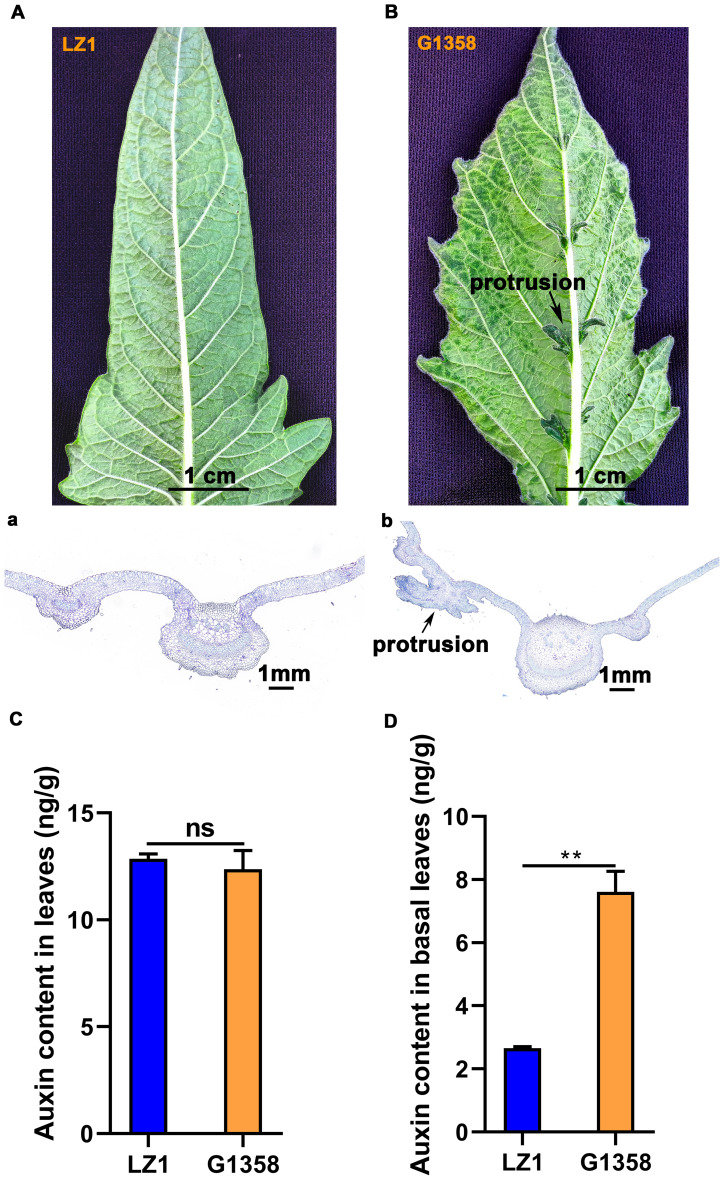
Comparison of leaf morphology and auxin content between G1358 and LZ1. **(A)** Leaf morphology of LZ1. **(B)** Leaf morphology of G1358. (a) Toluidine blue-stained leaves of LZ1. (b) Toluidine blue-stained leaves of G1358. The blue-green color represents the lignified cell wall, while the purple-blue color represents the cellulose cell wall. **(C)** Auxin content in the leaves of G1358 and LZ1. **(D)** Auxin content in the leaf base of G1358 and LZ1. Paired data were evaluated using Student’s *t*-test. ‘ns’ denotes no significant difference, while asterisks denote statistically significant differences (**p < 0.01). The bars indicate the mean ± SD from 3 independent biological experiments. .

There was an obvious difference in leaf morphology between G1358 and LZ1. The leaves of LZ1 had a smooth surface without protrusions, whereas G1358 leaves exhibited small protrusions. The field morphology of G1358 is shown in **Supplementary Figure S1**. Paraffin sectioning and toluidine blue staining allowed for detailed observation of these protrusions under a microscope (**Figures 1A, B**). Given the potential role of auxin in leaf morphology, we measured auxin content in both the entire leaves and the leaf bases (where the protrusions are located) of G1358 and LZ1. The levels of differential auxin metabolites between G1358 and LZ1 are presented in **Supplementary Figure S2**, **Supplementary Tables S2**, **S3**. We particularly focused on indole-3-acetic acid (IAA), which is known to exert a crucial role (Woodward and Bartel, 2005). There was no obvious difference in IAA content between the whole leaves of G1358 and LZ1 (**Figure 1C**). However, auxin content in the leaf base of G1358 was substantially higher compared to LZ1 (**Figure 1D**). These findings suggest that the observed leaf protrusions are not due to differences in auxin synthesis but rather to PAT, resulting in an uneven distribution of auxin.

### 3.2 Genome-wide assessment of ABCB, PIN, and AUX/LAX in S. indicum

PAT relies primarily on ABCB, PIN and AUX/LAX. To identify potential *ABCB, PIN, and AUX/LAX* genes in *S. indicum*, we obtained protein sequences for these families from *Arabidopsis thaliana* and *Oryza sativa* available in NCBI. After BLAST comparison and identification, 21 SiABCB family members, 11 SiPIN family members, and 5 SiLAX family members were identified in *S. indicum*. Genes were designated based on their chromosomal positions (**Supplementary Table S4**). SiABCB proteins are longer, with sequences spanning over 1000 amino acids, ranging from 1160 amino acids (SiABCB17, with a MW of 126.59 kDa) to 1709 amino acids (SiABCB8, with a MW of 184.14 kDa). Their theoretical pIs vary from 6.15 (SiABCB4) to 8.99 (SiABCB15). SiPIN proteins range from 390 amino acids (SiPIN11, with a MW of 42.75 kDa) to 651 amino acids (SiPIN5, with a MW of 70.74 kDa), with pIs varying between 7.4 (SiPIN10) and 10.35 (SiPIN11). The SiLAX proteins are approximately 500 amino acids long (ranging from 470 to 511). Their molecular weights (MWs) range from 52.95 kDa (SiLAX1) to 57.34 kDa (SiLAX3), and their theoretical-isoelectric-points (pIs) vary between 8.44 (SiLAX5) and 9.27 (SiLAX1). Subcellular localization prediction, generated using the WoLF PSORT tool, indicated that all SiABCB proteins, except SiABCB21 (which is located in the vacuole), were localized to the plasma membrane. For comprehensive details about gene IDs, chromosomal locations, gene loci, protein lengths, MWs, pIs, and subcellular locations, refer to **Supplementary Table S5**.

### 3.3 Phylogenetic analysis of ABCB, PIN, and LAX proteins in S. indicum, A. thaliana and O. sativa

**Figure 2:**
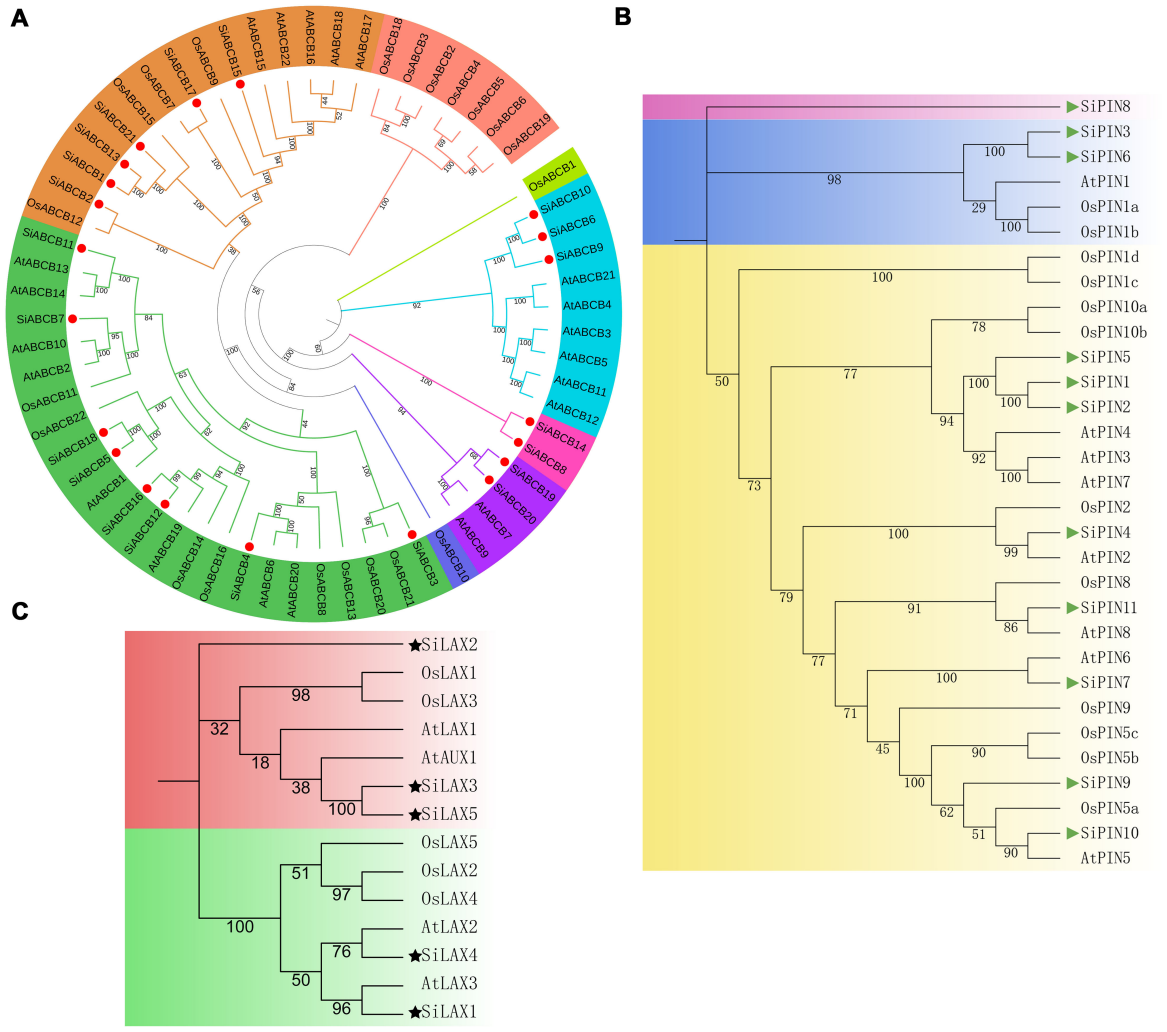
Phylogenetic analysis of the ABCB, PIN, and LAX protein families in *S. indicum*, *A. thaliana* and *O. sativa*. **(A)** Phylogenetic tree of 21 SiABCB, 21 AtABCB, and 21 OsABCB, with SiABCB marked by a dot. **(B)** Phylogenetic tree of 11 SiPIN, 8 AtPIN, and 12 OsPIN, with SiPIN represented by a triangle. **(C)** Phylogenetic tree of 5 SiLAX, 4 AtLAX/AUX and 5 OsLAX, with SiLAX indicated by a five-pointed star. Multiple-sequence-alignment was performed using ClustalW. The NJ tree was established in MEGA11 with 1000 bootstrap replicates. Distinct colors represent various groups of ABCB, PIN, and LAX proteins. .

Substantial advancements have been realized in understanding the diverse functions and regulatory mechanisms of auxin transporter gene families in *A. thaliana* (Mravec et al., 2009; Ding et al., 2012; Ung et al., 2022). The evolution and function of members of these gene family members vary across different plant species (Wang et al., 2018; Zhou et al., 2021). Investigating the evolutionary relationships of auxin transporters among closely related species, including *S. indicum*, *A*. *thaliana* and *O*. *sativa* will expand our knowledge of the biological roles of auxin transporter gene members in *S. indicum*. To this end, we constructed a neighbor-joining phylogenetic tree using 21 SiABCB, 11 SiPIN and 5 SiLAX from *S. indicum*; 21 AtABCB, 8 AtPIN and 4 AtLAX/AUX from *A*. *thaliana*; and 5 OsLAX, 12 OsPIN and 21 OsABCB from *O*. *sativa*. The protein sequences used were shown in **Supplementary Table S6**. ABCB genes were divided into eight groups, with SiABCBs clustering within five of these (**Figure 2A**). The PIN genes were grouped into 3 categories, with SiPINs showing greater homology to AtPINs than to OsPINs (**Figure 2B**). Sequence similarity and evolutionary patterns allowed us to classify the LAX/AUX genes into 2 main groups, with SiLAX1 and AtLAX3, as well as SiLAX4 and AtLAX2, showing relatively high homology. The other three LAX genes in *S. indicum* exhibited lower homology with those in *A. thaliana* and *O. sativa* (bootstrap < 50%; **Figure 2C**), although the SiLAX genes themselves displayed relatively high sequence similarity (**Supplementary Figure S3**). The sequence similarity of SiPINs and SiABCBs is presented in **Supplementary Figures S4**, **S5**, respectively, revealing partial domain sequence similarity.

### 3.4 Genomic architecture and conserved sequence patterns

The structural organization and conserved sequence patterns of genes serve as both the architectural foundation and evolutionary hallmark, providing valuable perspectives on the evolutionary changes in the gene family’s structure. As shown in **Supplementary Figure S6**, all *SiABCB*, *SiPIN*, and *SiLAX* genes display a combination of introns and exons. Except for SiABCB8, which contains 23 exons, the other genes consist of 4-12 exons (**Supplementary Table S7**). Members within the same cluster typically exhibit similar sequence characteristics, reflecting their close evolutionary relationships.

**Figure 3:**
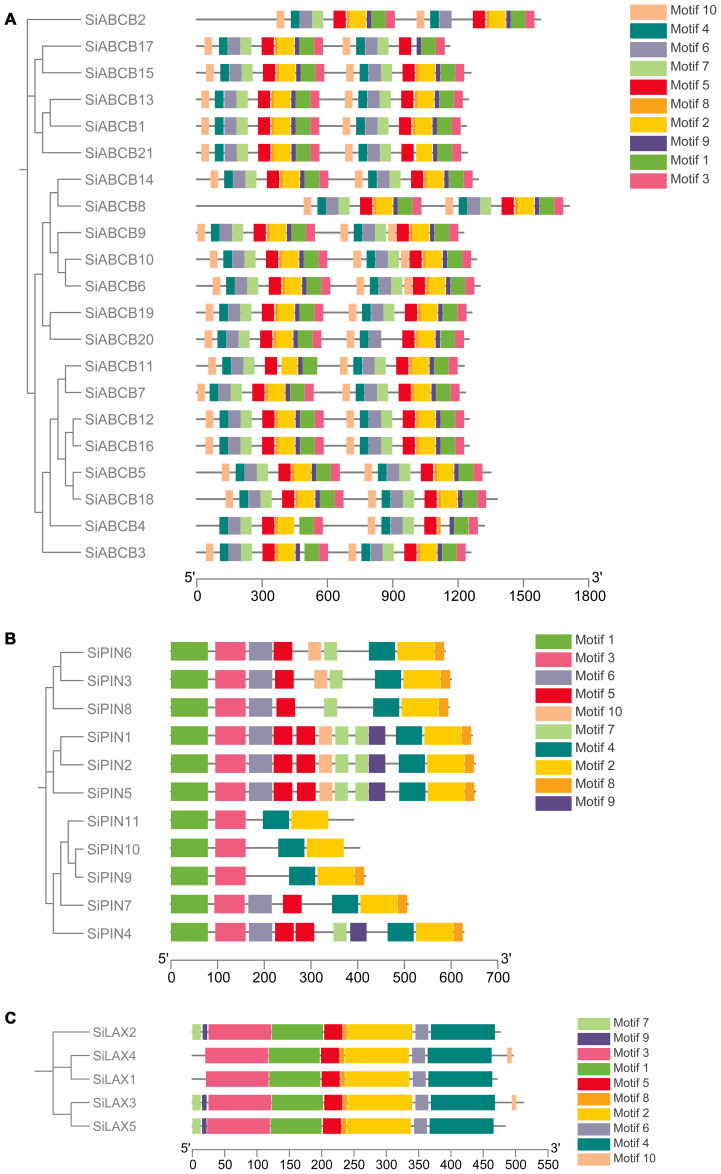
Analysis of conserved motifs in SiABCB, SiPIN, and SiLAX proteins. **(A)** Conserved motif analysis of SiABCB proteins. **(B)** Conserved motif analysis of SiPIN proteins. **(C)** Conserved motif analysis of SiLAX proteins. The *SiABCB, SiPIN*, and *SiLAX* genes are grouped according to phylogenetic analysis shown on the left. Ten distinct conserved motifs were estimated using MEME software, with the scale bar displayed at the bottom.

To explore the diversity in SiABCB, SiPIN, and SiLAX gene families, we performed a predictive assessment of conserved sequence motifs. Ten conserved motifs (Motifs 1 to 10) were identified, ranging from 8 to 100 amino acids in length (**Supplementary Figures S7**-**S9**). The protein sequences associated with these motifs are listed in **Supplementary Table S8**. *SiABCB* genes generally harbor more than 10 motifs (**Figure 3A**), while all *SiPIN* genes include Motifs 1 and 3 (**Figure 3B**). All *SiLAX* genes contain Motifs 1, 2, 3, 4, 5, 6, and 8 (**Figure 3C**). Additionally, proteins closely related within adjacent clades of the phylogenetic trees often display similar or identical motif compositions, indicating a strong association between motif architecture and evolutionary relationships.

### 3.5 Chromosome locations and duplications of SiABCB, SiPIN, and SiLAX

**Figure 4:**
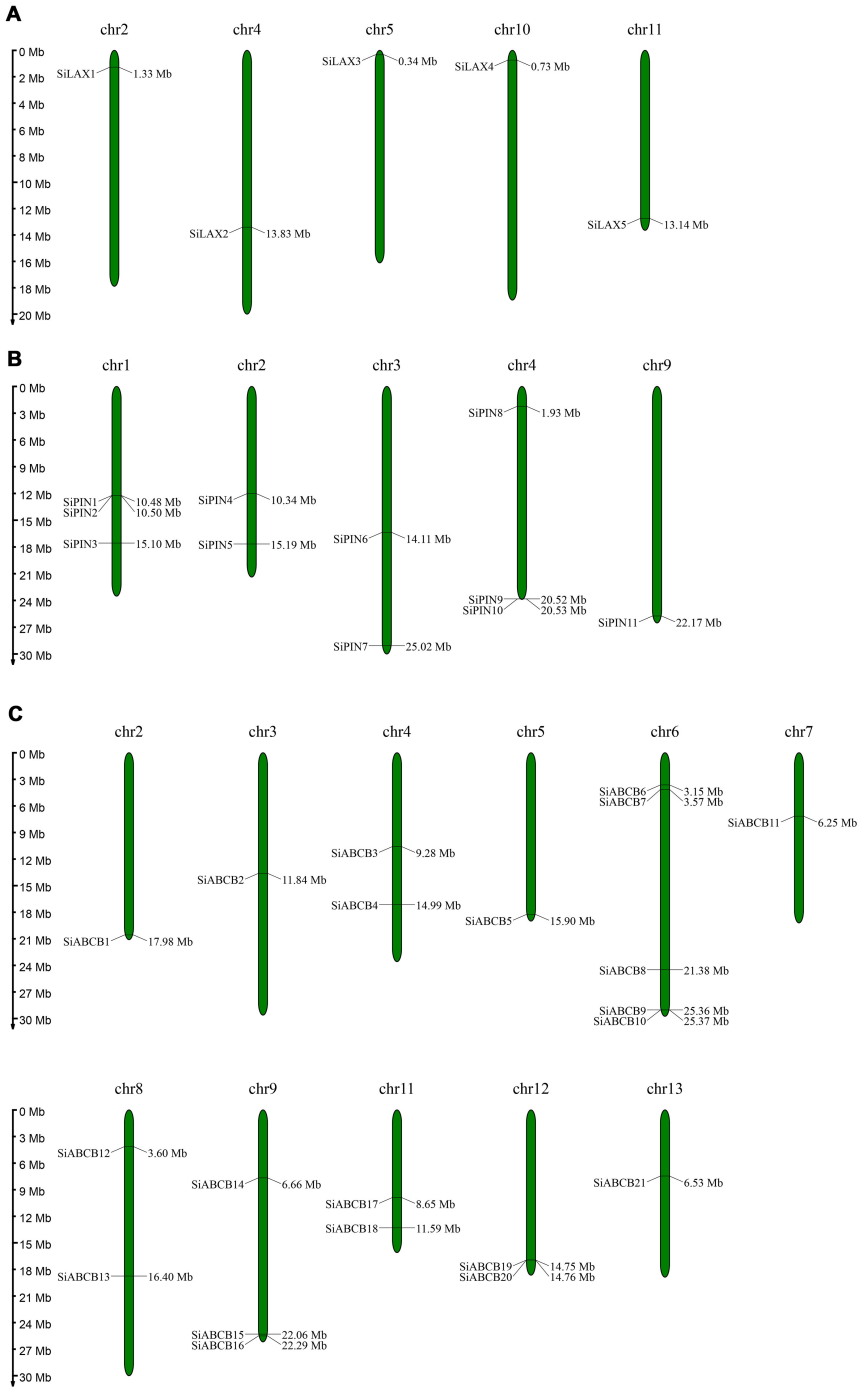
Chromosomal distribution of *SiABCB*, *SiPIN*, and *SiLAX* genes. Genes are annotated on the left side of the chromosomes, while position markers are displayed on the right. The scale bar on the left denotes chromosome lengths (Mb). **(A)** Chromosomal distribution of *SiLAX* genes. **(B)** Chromosomal distribution of *SiPIN* genes. **(C)** Chromosomal distribution of *SiABCB* genes.

The chromosomal localization of *SiABCB*, *SiPIN*, and *SiLAX* genes within the *S. indicum* genome was determined by analyzing chromosomal information and using MapChart for visualization. *SiABCB* genes were absent from chromosomes 1 and 10 but were found on all other chromosomes (**Figure 4A**), while the 11 *SiPIN* genes were distributed unevenly and non-randomly across five chromosomes (**Figure 4B**). The five *SiLAX* genes were each mapped to distinct chromosomes (**Figure 4C**). Notably, *SiABCB*, *SiPIN* and *SiLAX* genes were all present on chromosomes 2 and 4, with chromosome 6 exhibiting the greatest number of *SiABCB* genes (total = 5).

**Figure 5:**
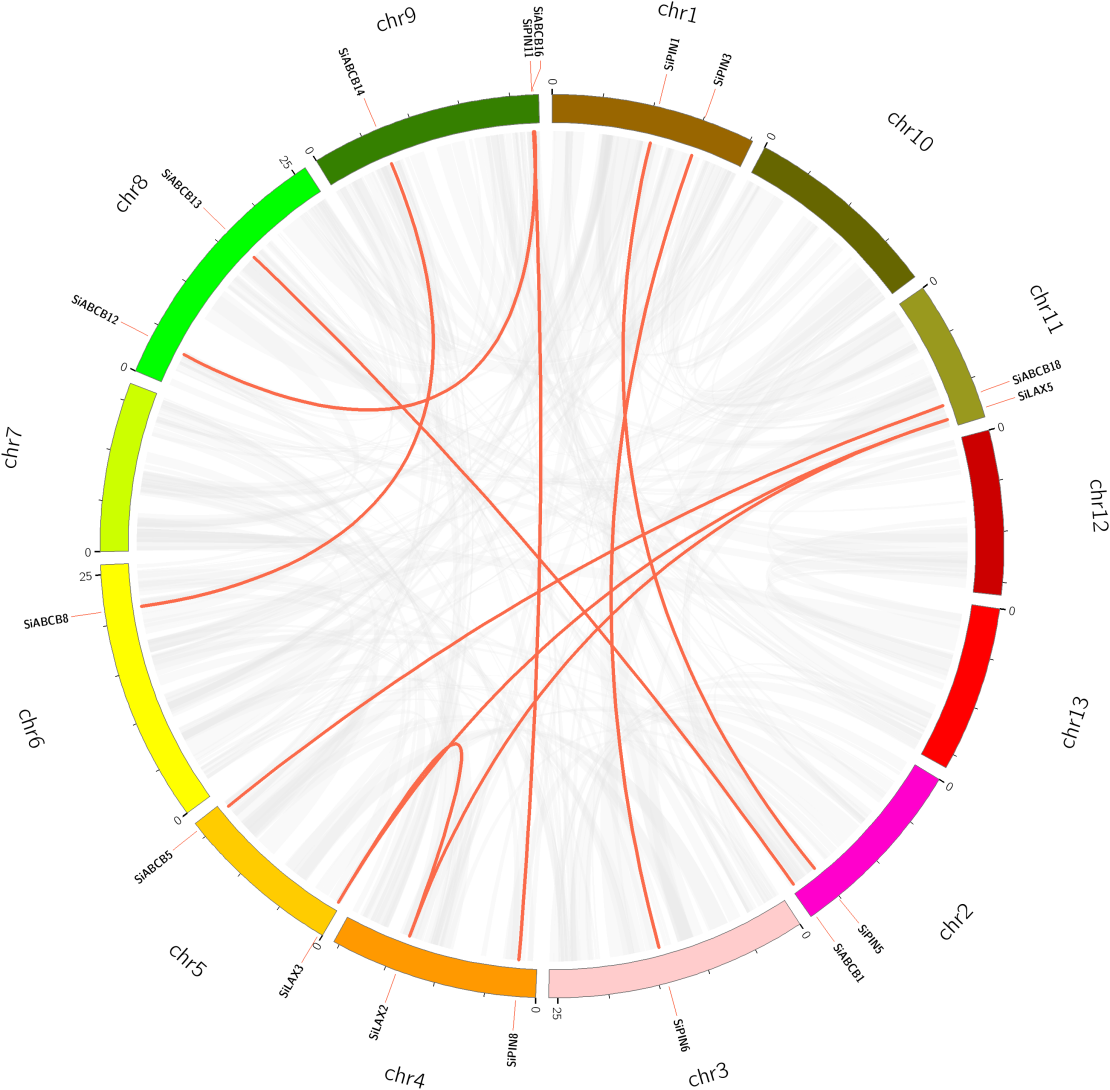
Distribution of *SiABCB, SiPIN*, and *SiLAX* gene pairs across chromosomes in the *S. indicum* genome. Chromosomes are represented by boxes, and homologous gene pairs by red lines.

Considering the critical role of gene duplication in the expansion of gene families during evolution, we conducted a gene duplication analysis within the *S. indicum* genome. No segmental duplications of *SiABCB*, *SiPIN*, and *SiLAX* genes were detected (**Figure 5**), suggesting that tandem duplications primarily facilitated the expansion of these gene families.

We also explored the collinearity between *S. indicum* and *A. thaliana*, identifying 27 collinear pairs of homologous genes between SiABCB/SiPIN/SiLAX and their *AtABCB*/*AtPIN*/*AtLAX* counterparts. In a similar analysis between *S. indicum* and *O. sativa*, only five pairs of homologous genes arranged in a collinear fashion were found between *SiLAX*/*SiABCB* and *OsLAX*/*OsABCB*. Notably, no homologous PIN genes were found between *S. indicum* and *O. sativa* (**Supplementary Figure S10** and **Supplementary Table S9**, **S10**).

The selection pressure on the duplication of *SiABCB*, *SiPIN*, and *SiLAX* genes was assessed using the *Ka/Ks* ratio (**Supplementary Table S11**). A *Ka/Ks* value less than 1 typically indicates purifying selection, a ratio of 1 denotes neutral evolution, and a value greater than 1 represents positive selection (Hu and Banzhaf, 2008). All *Ka/Ks* values were found to be < 1, suggesting that *SiABCB*, *SiPIN*, and *SiLAX* have undergone purifying selection throughout their evolutionary history.

### 3.6 Interaction network of SiABCB, SiPIN, and SiLAX proteins

A protein-protein interaction network was constructed to investigate the relationships among SiABCB, SiPIN, and SiLAX, and other proteins in *S. indicum* (**Supplementary Figure S11** and **Supplementary Table S12**). This network included 39 proteins, 29 of which belonged to the SiABCB, SiPIN, and SiLAX families, while the remaining 10 were from other protein families. This suggests that the functions of SiABCB, SiPIN, and SiLAX proteins may depend on their interactions with other proteins. Additionally, some SiABCB proteins were found to directly interact with each other.

### 3.7 Cis-element analysis of SiABCB, SiPIN, and SiLAX gene promoter regions in S. indicum

**Figure 6:**
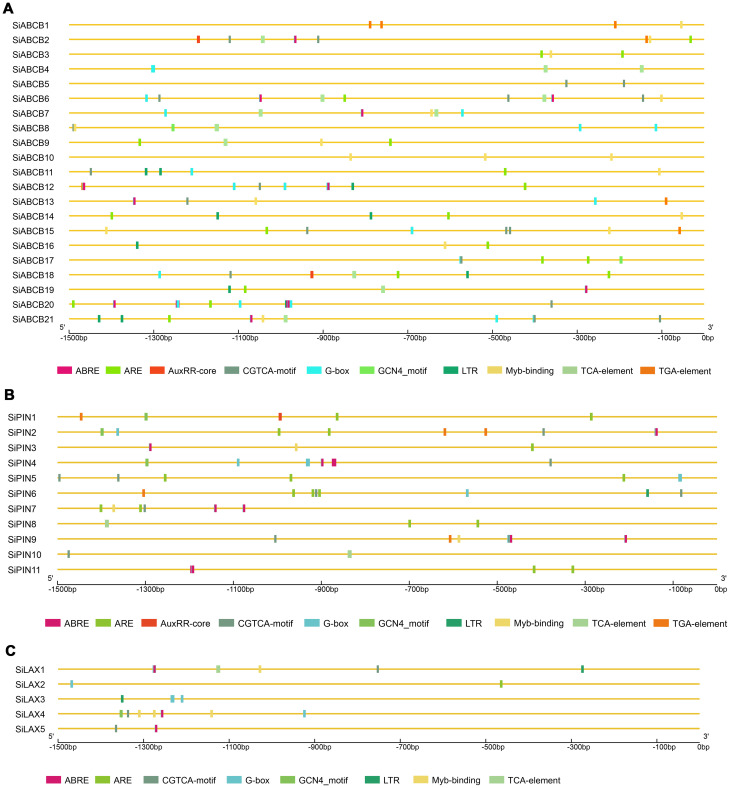
Cis-elements analysis in *SiABCB*, *SiPIN*, and *SiLAX* promoter regions. Different colored boxes at the bottom symbolize various cis-acting elements. **(A)** Cis-elements analysis in the promoter regions of *SiABCB* genes. **(B)** Cis-elements analysis in the promoter regions of *SiPIN* genes. **(C)** Cis-elements analysis in the promoter regions of *SiLAX* genes.

The diversity of gene expression under different conditions may be associated with the promoter region. To explore potential cis-regulatory elements in *SiABCB*, *SiPIN*, and *SiLAX* gene promoter regions, we assessed and compared the 1.5 kb upstream of the start codon (ATG) for each gene. Various cis-elements associated with light response, abiotic stress, plant hormone response, MYB binding and tissue-specific expression were determined (**Figure 6** and **Supplementary Table S13**). Specifically, the promoter region of SiLAX lacks auxin-responsive elements. However, elements responsive to hormones (auxin, abscisic acid, and salicylic acid), light, and low temperatures were the most abundant. This implies that PAT proteins may play significant roles in hormonal regulation and the response to diverse abiotic stresses in *S. indicum*.

### 3.8 Expression analysis of SiLAX, SiPIN and SiABCB

**Figure 7:**
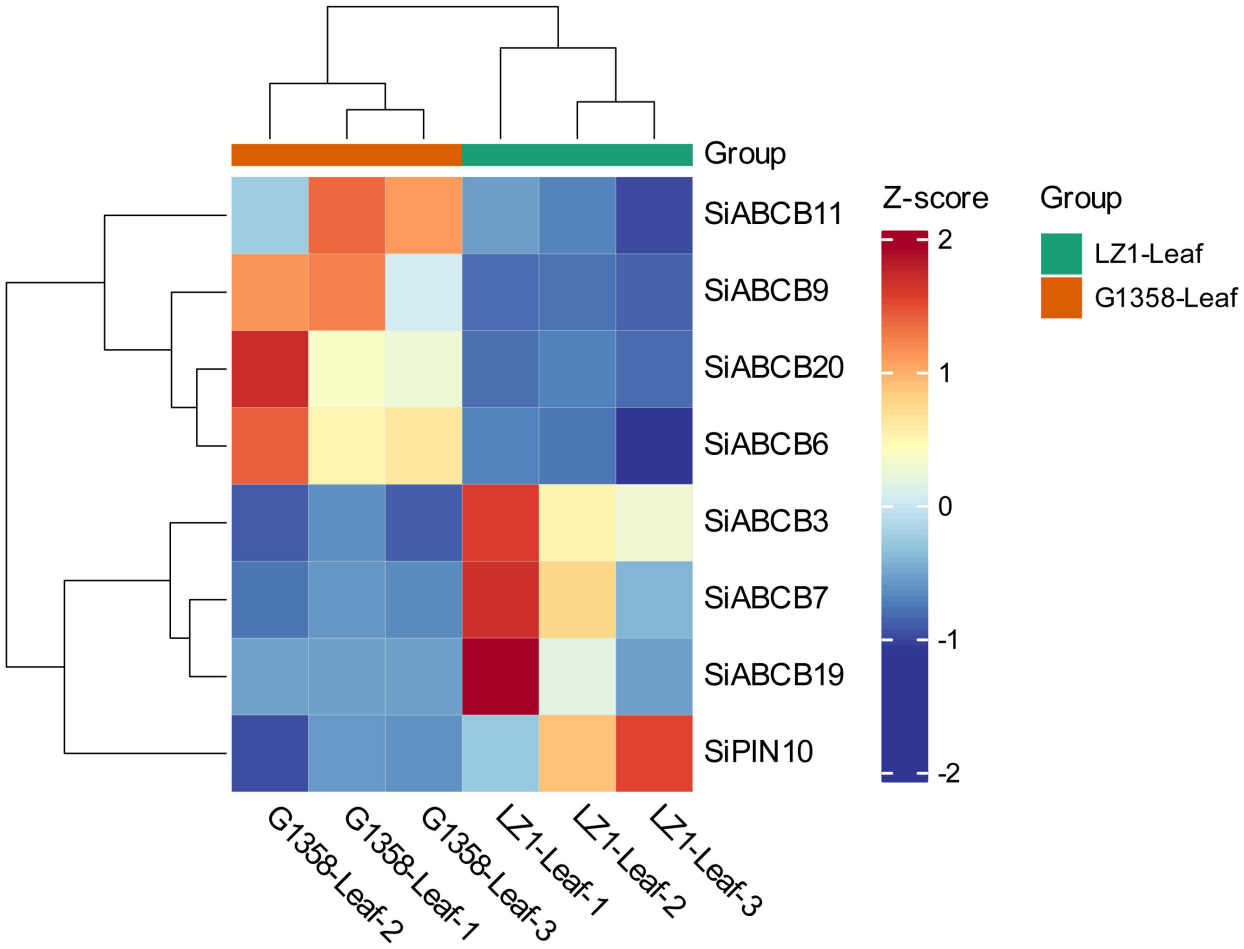
Enrichment of differentially expressed *SiABCB, SiPIN*, and *SiLAX* genes in the leaves of G1358 and LZ1. Heatmap blocks indicate gene expression levels, with red representing upregulation and blue representing downregulation, based on RNA-seq data.

**Figure 8:**
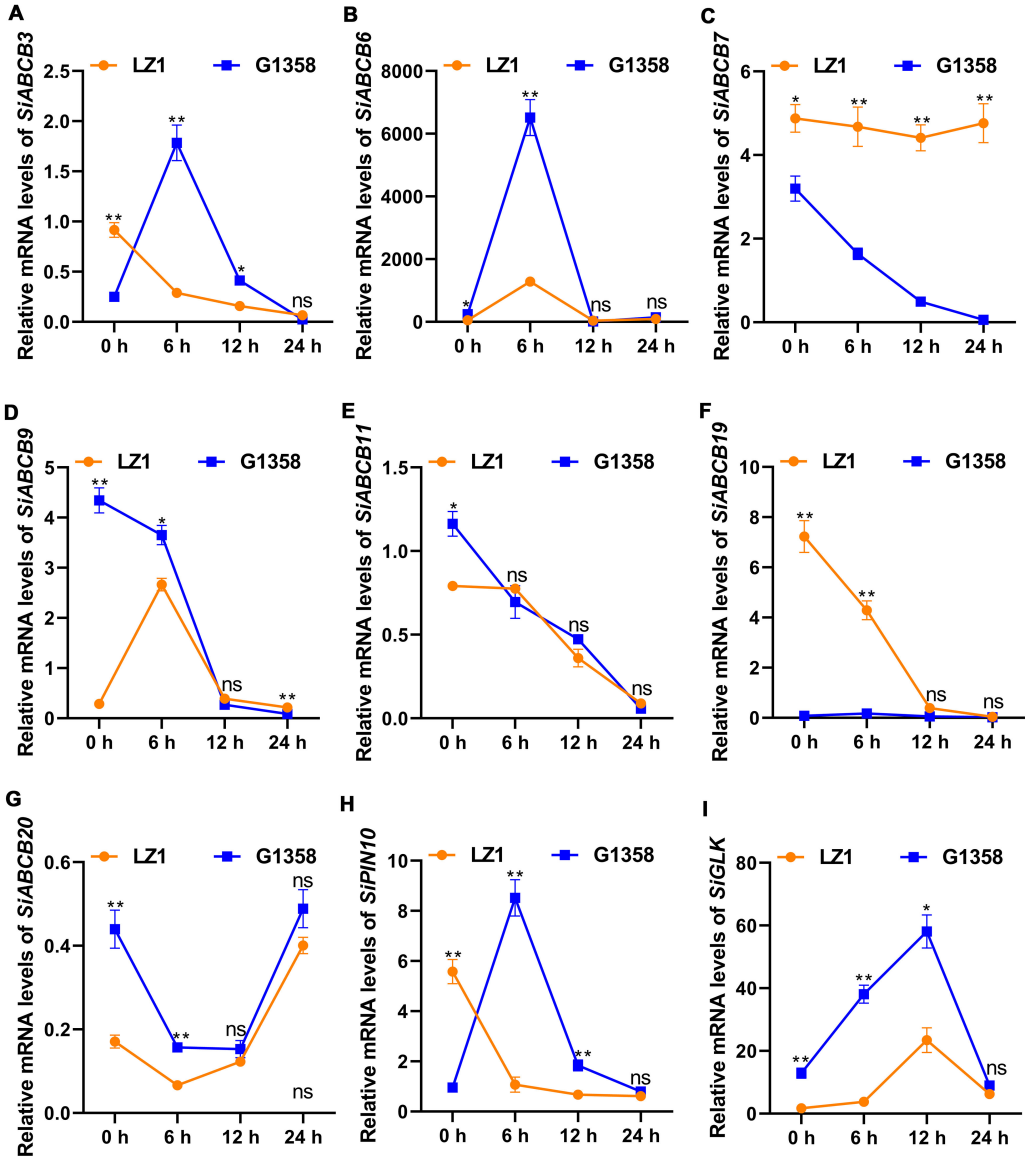
RT-qPCR results for the relative mRNA levels of DEGs in response to NAA treatment in the leaves of LZ1 and G1358. **(A–I)** Relative mRNA levels of *SiABCB3*, *SiABCB6*, *SiABCB7*, *SiABCB9*, *SiABCB11*, *SiABCB19*, *SiABCB20*, *SiPIN10*, and *SiGLK* were measured after treatment with 10 mg/L NAA for 0, 6, 12, and 24 h. *Actb* was utilized as the internal control. Paired data were evaluated using Student’s *t*-test. ‘ns’ denotes no significant difference, while asterisks denote statistically significant differences (*p < 0.05, **p < 0.01). The bars indicate the mean ± SD from 3 independent biological experiments.

A total of 4095 DEGs (differential expressed genes) were identified between the leaves of LZ1 and G1358, with 1,707 genes upregulated and 2,388 downregulated (**Supplementary Figure S12**). Among these, eight *SiABCB*, *SiPIN*, and *SiLAX* genes showed differential expression: *SiABCB6*, *SiABCB9*, *SiABCB11*, *SiABCB20* were upregulated in G1358 leaves, while *SiABCB3*, *SiABCB7*, *SiABCB19*, *SiPIN10* were downregulated. A heatmap depicting the expression of these eight genes in G1358 and LZ1 leaves was generated following RNA-seq analysis (**Figure 7**). To examine the regulatory effects of auxin on these DEGs, 10 mg/L of naphthylacetic acid (NAA) was applied to the leaves during the seedling stage, and mRNA expression analysis was conducted. The results indicated that, without NAA (0 h), the mRNA levels of *SiABCB9*, *SiABCB11* and *SiABCB20* were upregulated in G1358 compared to LZ1, while those of *SiABCB3*, *SiABCB7*, *SiABCB19* and *SiPIN10* were downregulated in G1358 compared to LZ1. The expression profiles aligned with the RNA-seq data, confirming its accuracy and reliability. In G1358, NAA treatment significantly upregulated the expression of *SiABCB3*, *SiABCB6* and *SiPIN10*. Conversely, *SiABCB7* and *SiABCB9* may play inhibitory roles in PAT in G1358 compared with LZ1 (**Figures 8A–H**).

The GARP-type transcription factor (GLK) gene family plays a crucial role in plant development, particularly in the regulation of chloroplast biogenesis and photosynthesis (Fitter et al., 2002). *GLK* genes encode transcription factors that are essential for the proper development and function of chloroplasts (Zhang et al., 2024b). We found that there was only one *GLK* gene in *S. indicum*. Phylogenetic analysis revealed that *SiGLK*, the sole *GLK* gene in *S. indicum*, is closely related to *AhGLK* (**Supplementary Figure S13** and **Supplementary Table S14**). We can further understand the regulation mechanism of G1358 sesame leaf protrusions formation and photosynthetic efficiency enhancement by analyzing the expression of *GLK* gene through RT-qPCR. We found that *SiGLK* expression was much higher in G1358 than in LZ1, and NAA application significantly increased *SiGLK* expression (**Figure 8I**).

**Figure 9:**
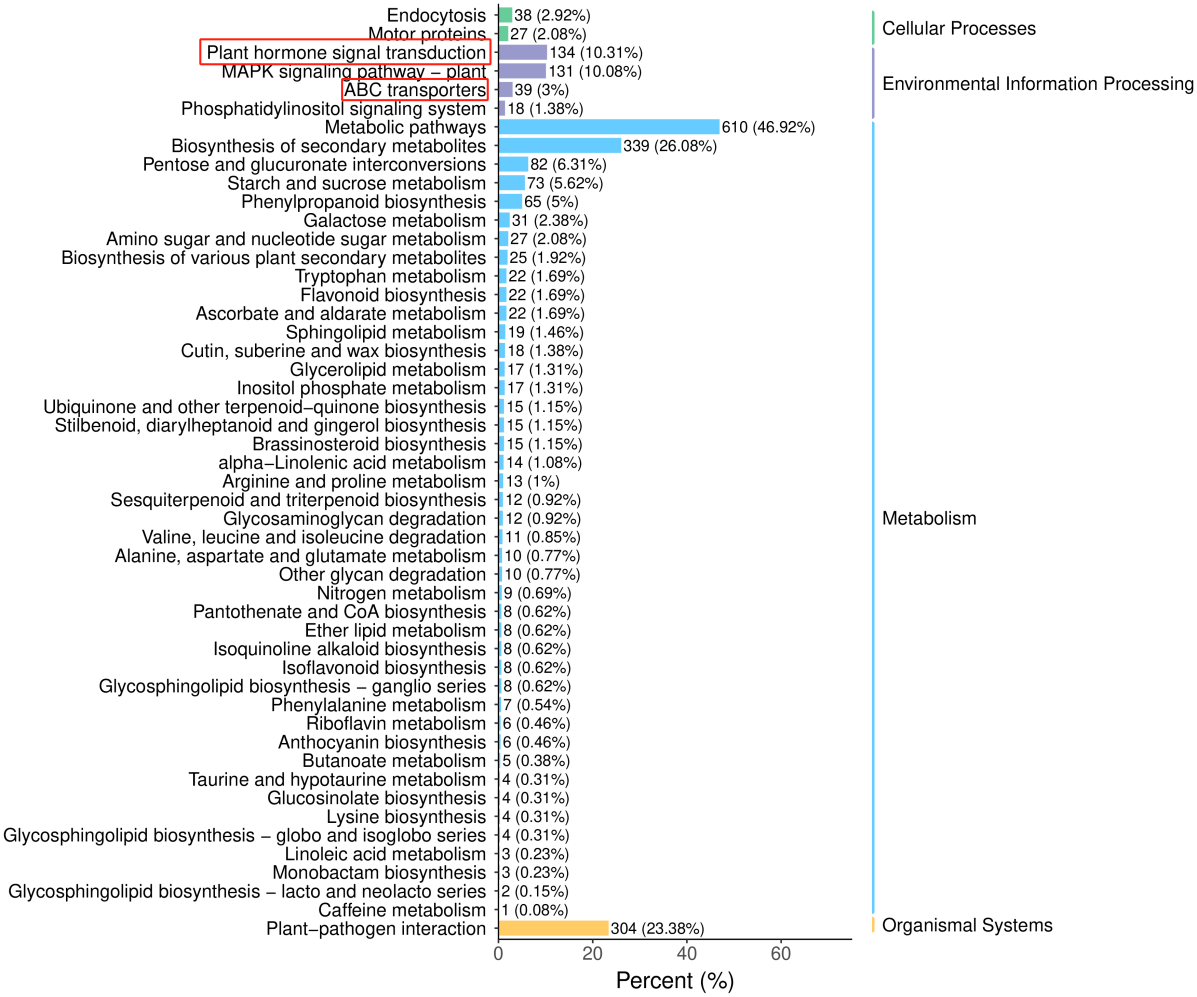
KEGG pathway classification analysis of DEGs in G1358 and LZ1.

KEGG analysis of the DEGs identified 133 enriched pathways in the G1358-Leaf vs. LZ1-Leaf comparison (**Supplementary Table S15**). The top 10 pathways included plant hormone signal transduction and ABC transporters (**Figure 9**). SiABCB20, a gene associated with the ABC transporter signaling pathway, was highly enriched in G1358 (**Supplementary Figure S14**). These findings suggest that in G1358, auxin may positively regulate PAT through the actions of *SiABCB3*, *SiABCB6*, and *SiPIN10*, while *SiABCB7* and *SiABCB9* may play a negative regulatory role in this process.

### 3.9 SPAD, Pn and CAP values

**Figure 10:**
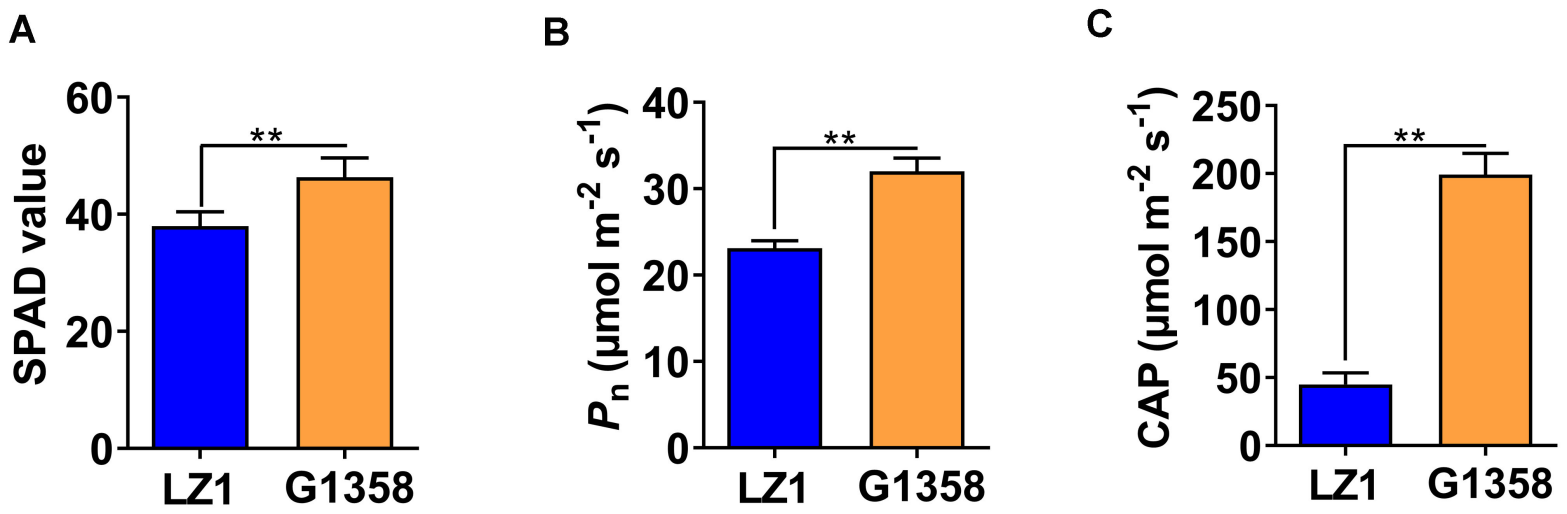
Comparison of relative chlorophyll content and photosynthetic rate between LZ1 and G1358. **(A)** SPAD values of LZ1 and G1358. **(B)** Net photosynthetic rates (Pn) of LZ1 and G1358. **(C)** Canopy photosynthetic rate (CAP) of LZ1 and G1358. Experiments were performed in triplicate, and statistical differences were detected with Student’s *t*-test (**p < 0.01). Bars represent mean ± SD.

To assess the impact of leaf shape, specifically the leaf protrusions observed in G1358, on plant photosynthesis, we measured the SPAD, Pn, and CAP values in both G1358 and LZ1 plants. The results indicated that G1358 exhibited higher SPAD, Pn, and CAP values compared to LZ1 (**Figure 10**), suggesting that the small protrusions on the leaves may significantly enhance the photosynthetic efficiency of the plants.

## 4 Discussion

PAT is a vital process in plant growth and development, impacting various aspects such as cell division, differentiation, and organ formation. PAT primarily involves the directional movement of auxins from their synthesis sites to other parts of the plant. This movement is essential for regulating growth patterns and organ formation (Goldsworthy and Rathore, 1985). The overall control of growth in plant tissues, including leaves, relies heavily on the balance and distribution of growth regulators like auxins. We identified a total of 21 SiABCBs, 11 SiPINs, and 5 SiLAXs in *S. indicum*. Notably, the expression levels of *SiABCB3*, *SiABCB6*, and *SiPIN10* were significantly upregulated under NAA treatment in the leaves of G1358, whereas *SiABCB7* and *SiABCB9* exhibited downregulation. These findings suggest that *SiABCB3*, *SiABCB6*, and *SiPIN10* play positive regulatory roles in PAT, thereby promoting the formation of leaf protrusions in G1358. This observation aligns with established theories regarding the involvement of PAT in plant morphogenesis (Wang et al., 2011). The elevated expression of *SiABCB3* and *SiABCB6* may have enhanced the polar transport of auxin, resulting in increased auxin concentrations at the leaf base. This accumulation could stimulate cell proliferation and contribute to the development of protrusions. Furthermore, these structural changes may improve photosynthetic efficiency by expanding the leaf surface area and enhancing light capture capacity (Traas, 2018). Our research aimed to elucidate the functions of the ABCB, PIN, and AUX/LAX gene families in regulating leaf protrusion formation and photosynthetic performance in sesame. By providing insights into these mechanisms, this study offers a theoretical foundation for strategies aimed at improving crop yield through targeted genetic modifications.

### 4.1 The evolution and function of ABCB gene family

*ABCB* genes are responsible for diverse biological processes, including the transport of phytohormones such as auxin, which is crucial for plant growth and development (Dhaliwal et al., 2014; Okamoto et al., 2016). Studies across different plant species reveal both structural conservation and notable variations among *ABCB* genes. For example, the genomic copies of *ABCB1* differ between monocots and dicots, with variations in intron size and number that may affect gene expression and function (Dhaliwal et al., 2014). Similarly, the structure of *ABCB19* in *Arabidopsis thaliana* suggests its involvement in processes such as cytoplasmic streaming and gravitropism, reflecting specific adaptations of this transporter to environmental stimuli (Okamoto et al., 2016). In our research, we identified that *SiABCB* genes generally contain more than 10 motifs, with *SiABCB8* having 23 exons, indicating potentially complex functions that warrant further investigation. Despite their classification as *ABCBs*, these genes exhibit functional differences: *SiABCB3* and *SiABCB6* were found to positively regulate PAT, whereas *SiABCB7* and *SiABCB9* negatively regulate PAT. Previous studies in rice and *Arabidopsis* have shown that tandem and segmental duplications are involved in the expansion of the ABC gene family (Xing et al., 2012). In our study, we identified 21 *SiABCB* genes, which matches the number found in both *A. thaliana* and *O. sativa*. Tandem duplications were implicated in the expansion of the SiABCB gene family in *S. indicum*. These findings suggest that *ABCB* genes have undergone significant evolutionary changes, potentially driven by environmental pressures or adaptation to new ecological niches.

### 4.2 The evolution and function of PIN gene family

PIN genes are essential for regulating PAT, a process essential for plant growth, development, and response to environmental stresses. The PIN gene family has experienced considerable evolution across various plant lineages. In common wheat (*Triticum aestivum* L.), a genome-wide analysis identified 44 *TaPIN* genes, which were classified into seven groups based on phylogenetic analysis (Kumar et al., 2021). Similarly, in *Solanum tuberosum*, PIN proteins were categorized into 14 distinct clades, tracing lineage to the common ancestor of green algae (Yang et al., 2019), indicating a rapid expansion of the PIN gene family in angiosperms compared with algae. In *Glycine max*, 23 *GmPIN* genes were identified, with evidence of segmental duplication events contributing to their diversity (Liu and Wei, 2017). In our study, we identified 11 *SiPIN* genes, which were classified into three groups. Notably, *SiPIN* genes show higher homology with *AtPIN* genes than with *OsPIN* genes. Tandem duplication events were found to drive the expansion of the SiPIN gene family. These findings collectively imply that the PIN gene family has undergone significant evolutionary changes, particularly in more complex plant lineages such as angiosperms.

### 4.3 The evolution and function of AUX/LAX gene family

The AUX/LAX family is crucial for mediating auxin entry into cells, which is fundamental for establishing the auxin gradients required for various developmental processes (Swarup and Peret, 2012; Swarup and Bhosale, 2019). The distinct expression patterns and functional diversification within the AUX/LAX family suggest that each member may contribute uniquely to processes such as root gravitropism, lateral root development, and leaf phyllotaxis (Swarup and Peret, 2012). Evolutionary analysis of the AUX/LAX gene family indicates subfunctionalization based on distinct spatial expression patterns and the inability of LAX sequences to rescue aux1 mutant phenotypes (Péret et al., 2012). This evolutionary process has resulted in the development of unique regulatory mechanisms for each gene within the family. Furthermore, evolutionary studies of the Aux/IAA family in plants reveal dual origins and variable nuclear localization signals, reflecting a complex evolutionary history of auxin signaling pathways (Wu et al., 2017). In *S. indicum*, five LAX genes with high sequence similarity were identified. Notably, SiLAX1 and AtLAX3, as well as SiLAX4 and AtLAX2, exhibit high homology, suggesting potential functional similarities. However, SiLAX was not identified among the differentially expressed genes in the G1358 and LZ1 transcriptomes, and no auxin-responsive elements were observed in the SiLAX promoter region. This suggests that SiLAX may not be involved in leaf protrusion formation in *S. indicum*.

### 4.4 Relationship between leaf morphology and photosynthetic rate

Evidence indicates that the geometric arrangement of the leaf lamina relative to light rays significantly impacts the photosynthetic rate (An) under oblique illumination conditions (Nikolopoulos et al., 2024). This suggests that the physical structure of the leaf, including its orientation with respect to light, plays a crucial role in optimizing photosynthesis. Our research demonstrated that small leaf protrusions can significantly enhance photosynthetic rates. The high expression of *SiGLK* increased the chlorophyll content and photosynthetic rate in *S. indicum*. The mechanisms underlying this increase are complex and warrant further investigation. In summary, leaf morphology, including its structural features, has a substantial effect on photosynthetic efficiency. These findings highlight the importance of considering leaf morphology in strategies aimed at improving photosynthesis in agricultural and environmental contexts.

Our comprehensive study of the SiABCB, SiPIN, and SiLAX gene families in *S. indicum* has provided detailed insights into their basic characteristics, chromosomal distribution, phylogeny, co-expression networks, gene structures, and subcellular localizations. The genome-wide investigation of ABCB, PIN, and AUX/LAX gene families in *S. indicum* reveals a complex interplay of auxin transport mechanisms that likely contribute to the development of leaf protrusions. Through auxin metabolite analysis and transcriptomic studies of sesame varieties G1358 and LZ1, we found evidence suggesting that PAT family genes are responsible for the formation of leaf protrusions in G1358. Expression pattern analysis revealed that *SiABCB3*, *SiABCB6*, and *SiPIN10* positively regulate PAT, whereas *SiABCB7* and *SiABCB9* negatively regulate PAT. By integrating findings from various plant species and focusing on the specific roles of these transporter families, this study enhances our understanding of auxin-mediated developmental processes in *S. indicum*. Our research has unveiled the significant roles of *ABCB*, *PIN*, and *AUX/LAX* genes in shaping sesame leaf morphology and influencing photosynthesis. However, the exact molecular mechanisms through which these genes modulate auxin transport remain to be further explored. Future research should aim to elucidate the specific genetic interactions and regulatory networks involving these auxin transporters in leaf protrusion formation, potentially providing insights into broader developmental principles applicable across the plant kingdom.

## Data Availability

The original contributions presented in the study are publicly available. This data can be found here:NCBI, PRJNA1215689.
