# Supplementary material for: Genome-wide investigation of ABCB, PIN, and AUX/LAX gene families and their involvement in the formation of leaf protrusions in Sesamum indicum
Source: Front Plant Sci. 2025 Jan 31;15:1526321. doi: 10.3389/fpls.2024.1526321 (PMC11825473; doi:10.3389/fpls.2024.1526321)
Supplement: Supplementary file 1 [file DataSheet1.docx]

**Figure supplements**


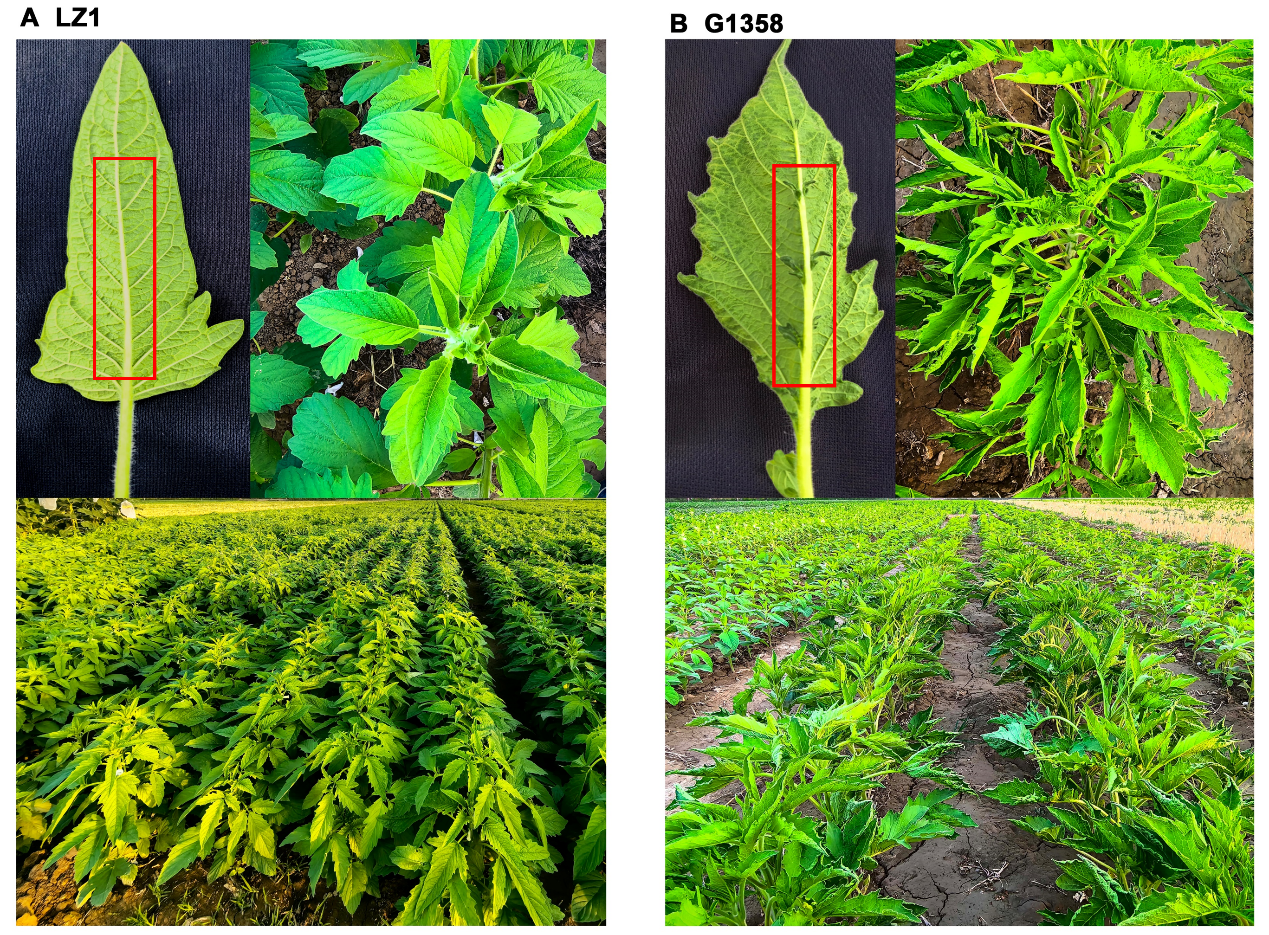


**Figure S1.** Morphology of G1358 in the field. The red box represents the leaf base.


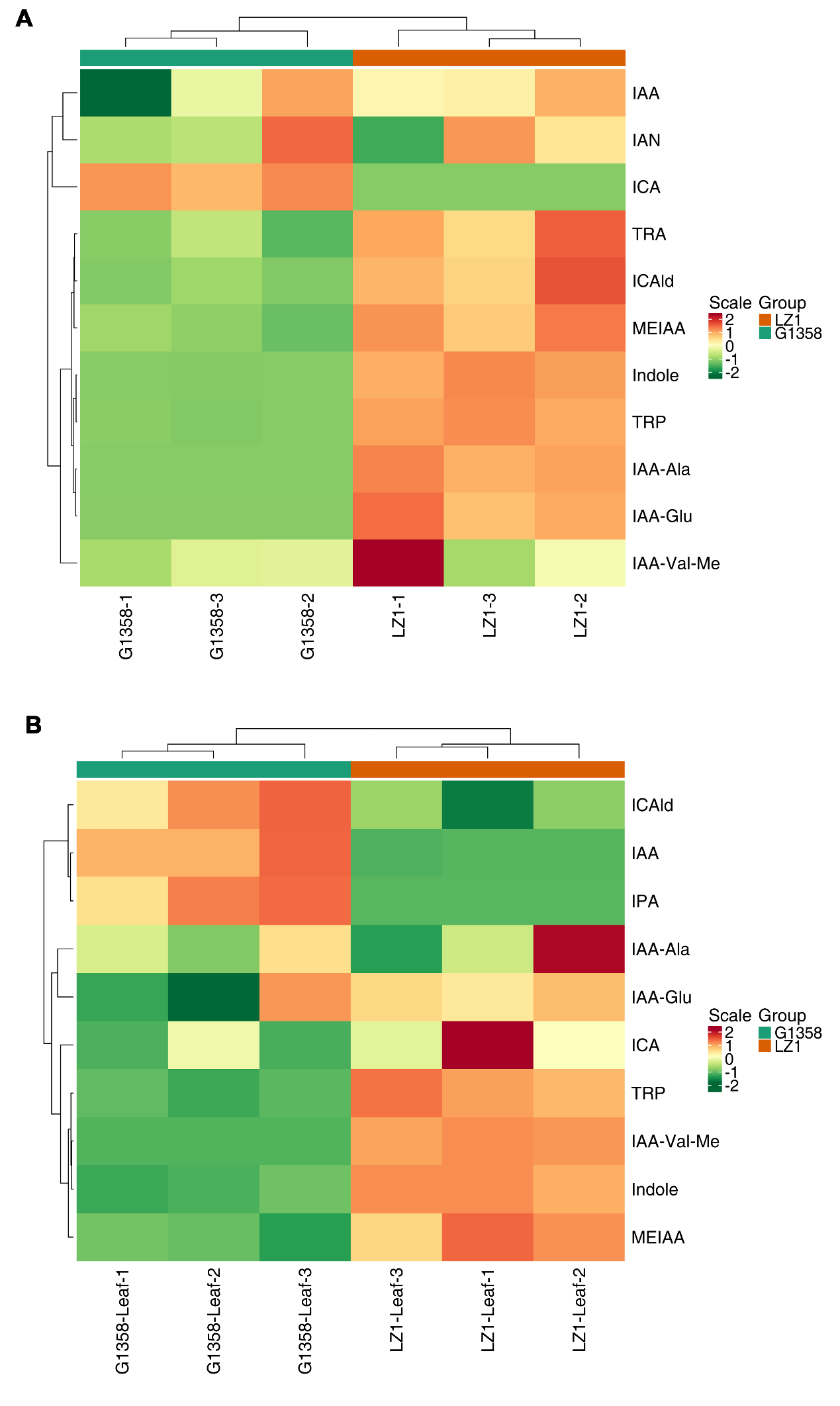


**Figure S2.** The clustering of auxin metabolites and significantly different metabolites between G1358 and LZ1. **(A)** A heat map showing auxin metabolite levels in the whole leaves of G1358 and LZ1. **(B)** A heat map showing auxin metabolite levels in basal leaves of G1358 and LZ1. Different colors represent different values of auxin metabolites with different contents after standardized treatment (red represents high content, green represents low content).


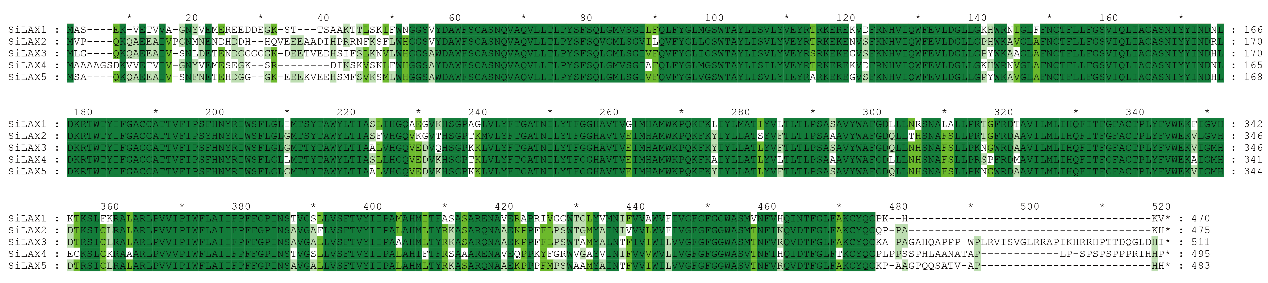
 **Figure S3.** Multiple sequence alignment of SiLAX subfamily proteins using Genedoc software.


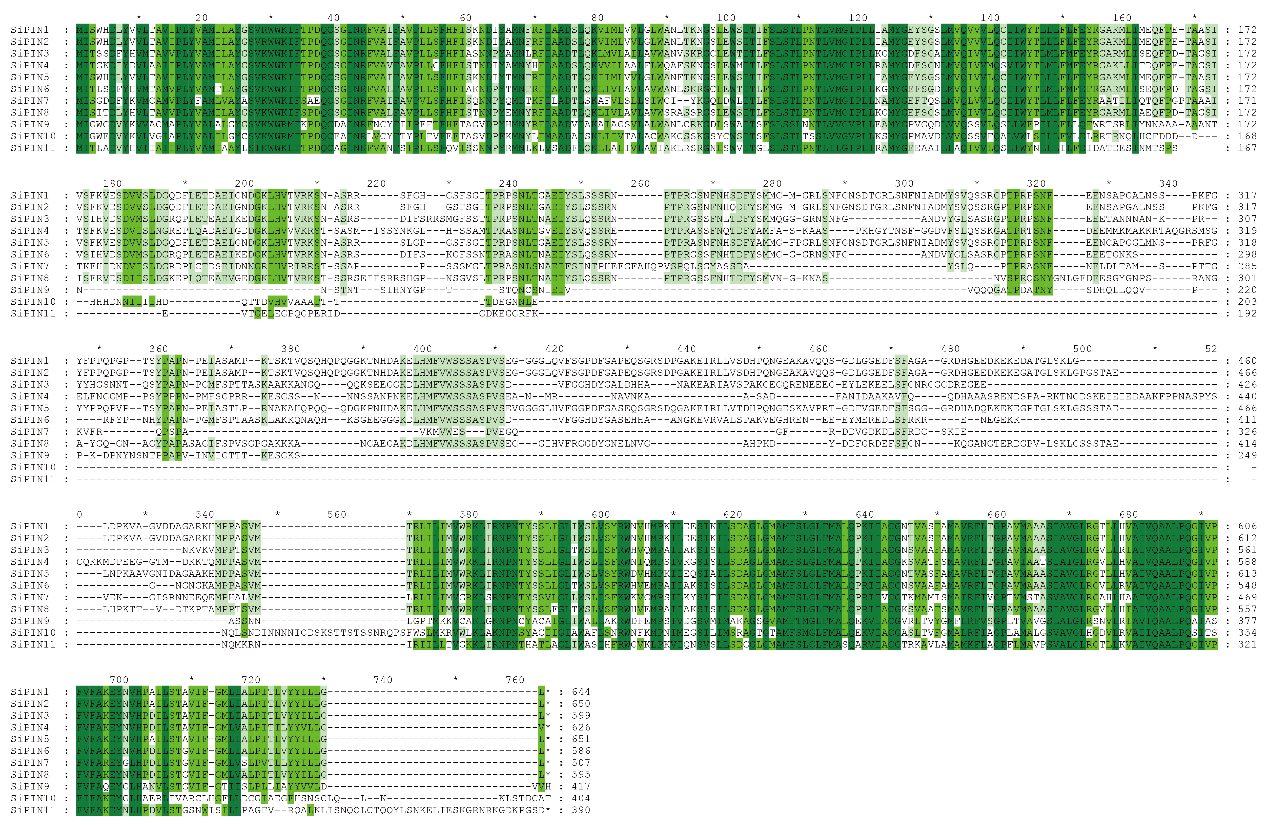


**Figure S4.** Multiple sequence alignment of SiPIN subfamily proteins using Genedoc software.


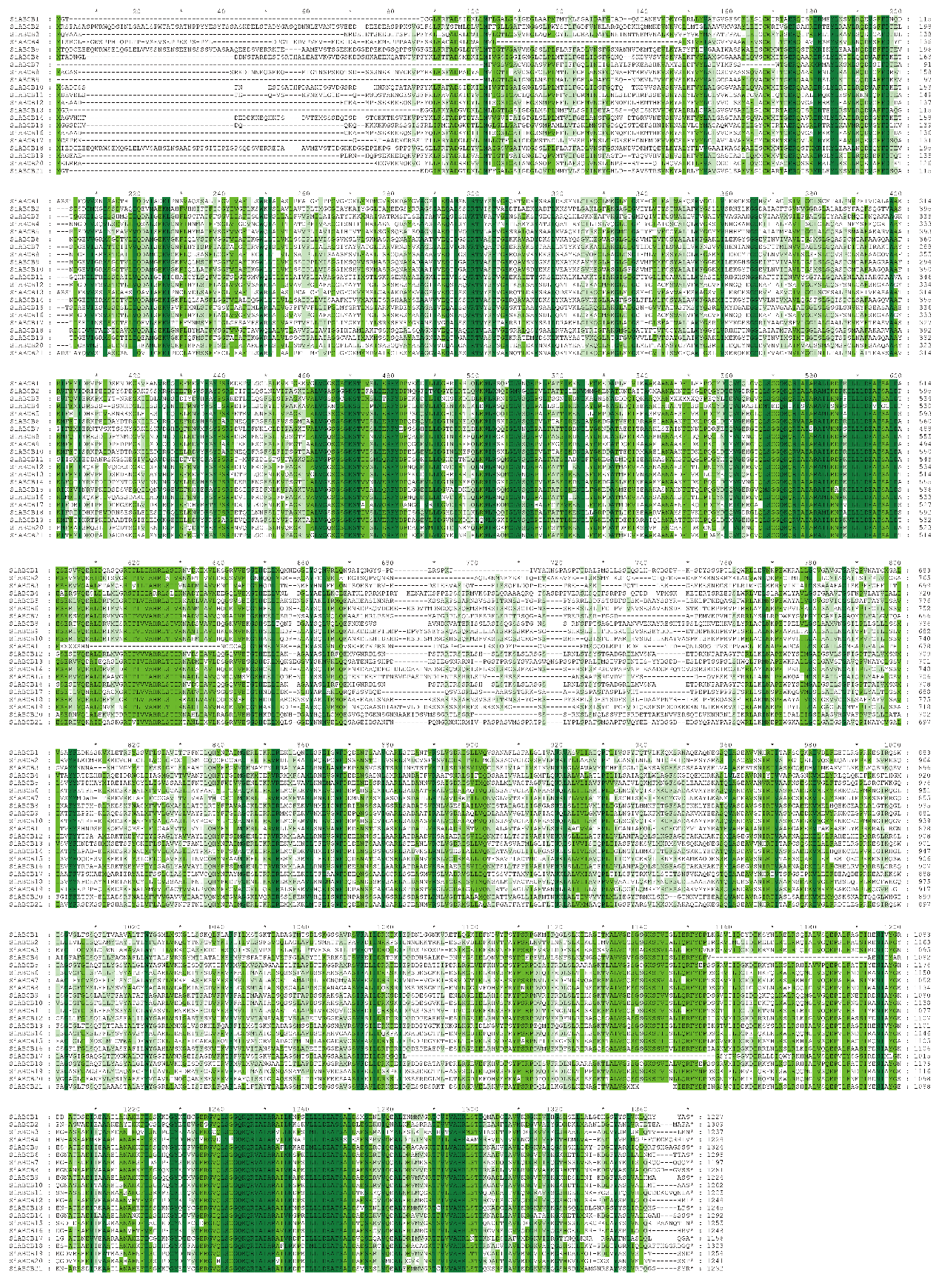


**Figure S5.** Multiple sequence alignment of SiABCB subfamily proteins using Genedoc software.


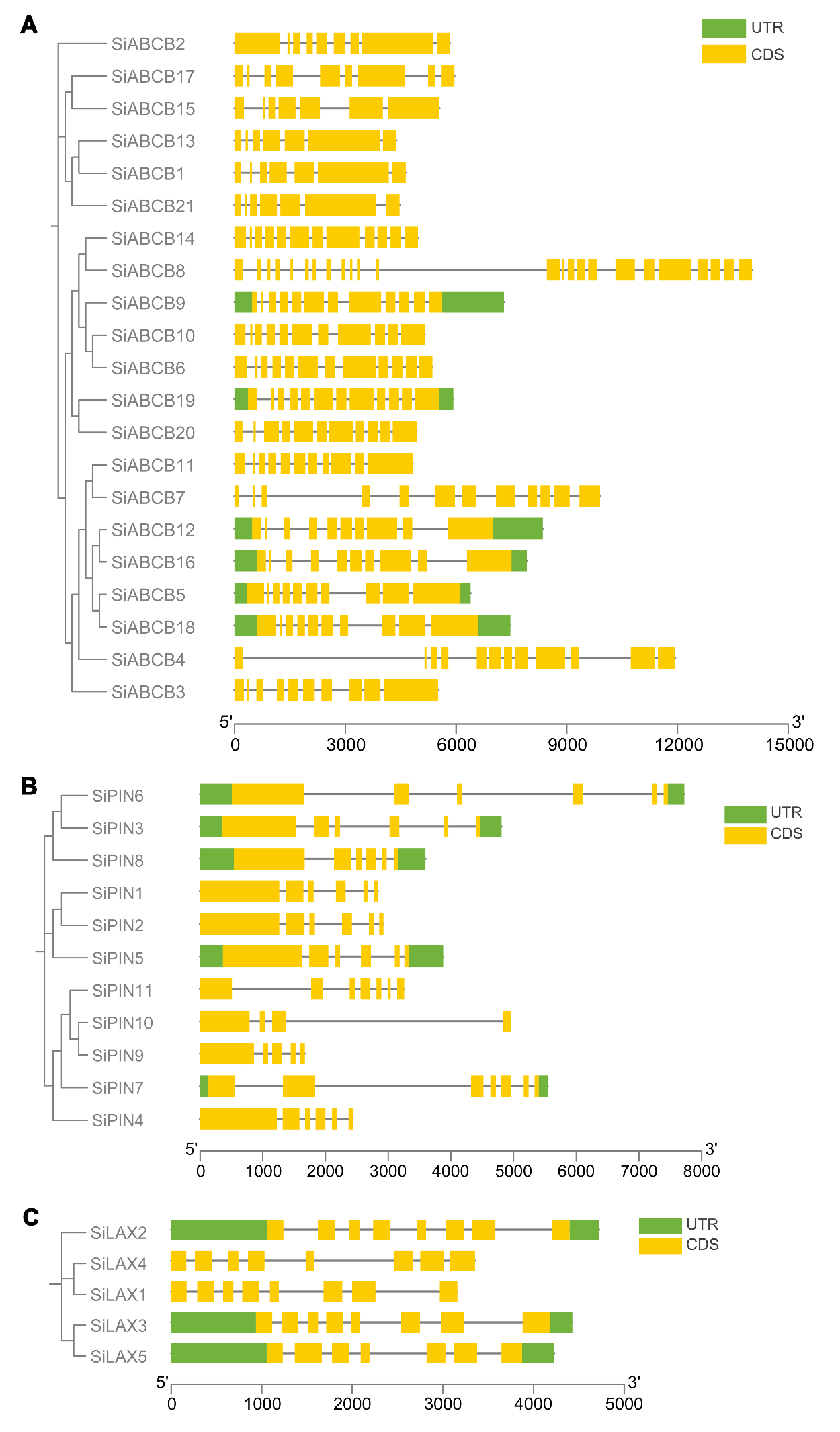


**Figure S6.** The gene structure analysis of *SiABCB/SiPIN/SiLAX*. **(A)** The gene structure analysis of *SiABCB* genes using the tool of GSDS. **(B)** The gene structure analysis of *SiPIN* genes using the tool of GSDS. **(C)** The gene structure analysis of *SiLAX* genes using the tool of GSDS. Green rectangles indicate UTR, grey lines indicate intron, and orange rectangles indicate exon.


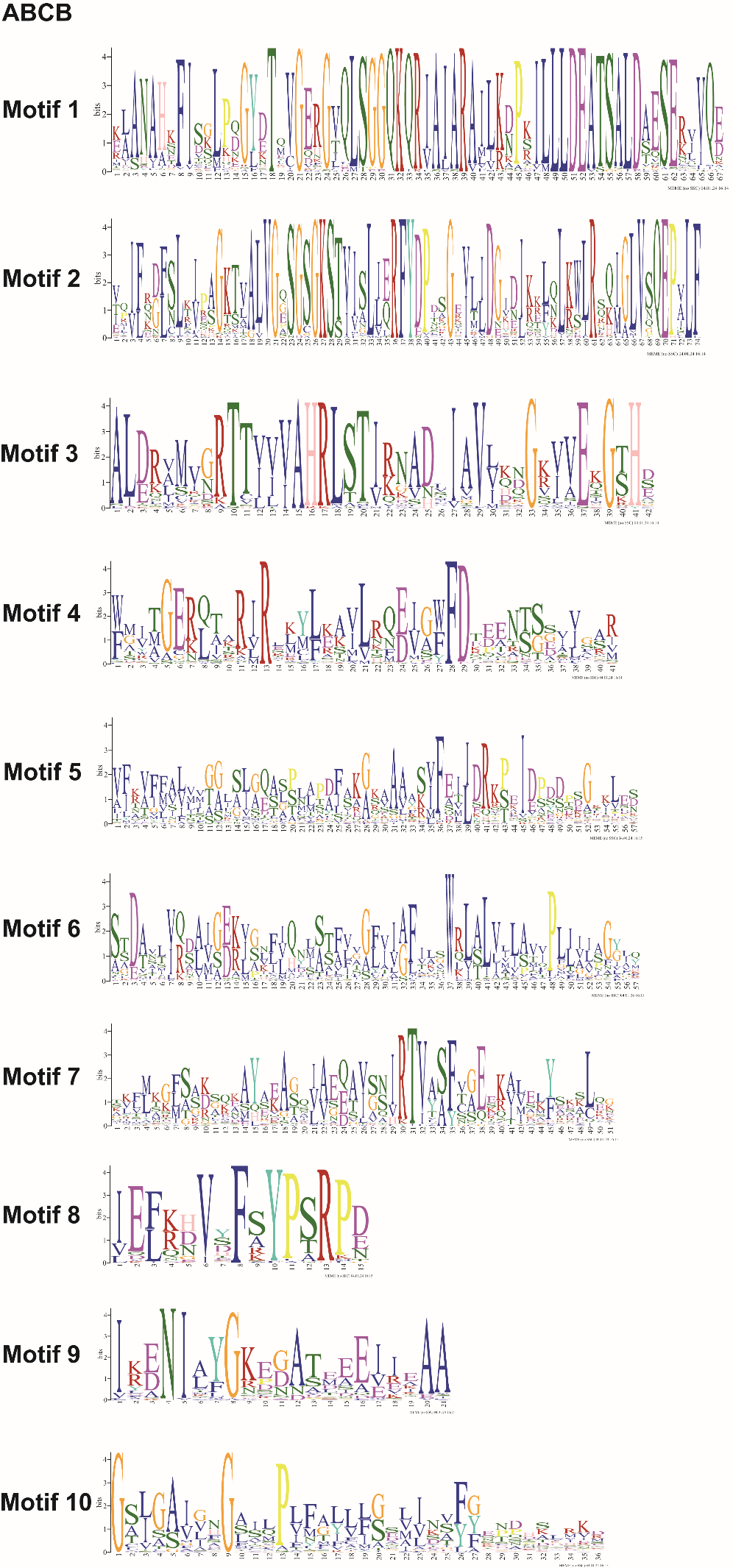


**Figure S7.** Ten main conserved motifs of SiABCB proteins.


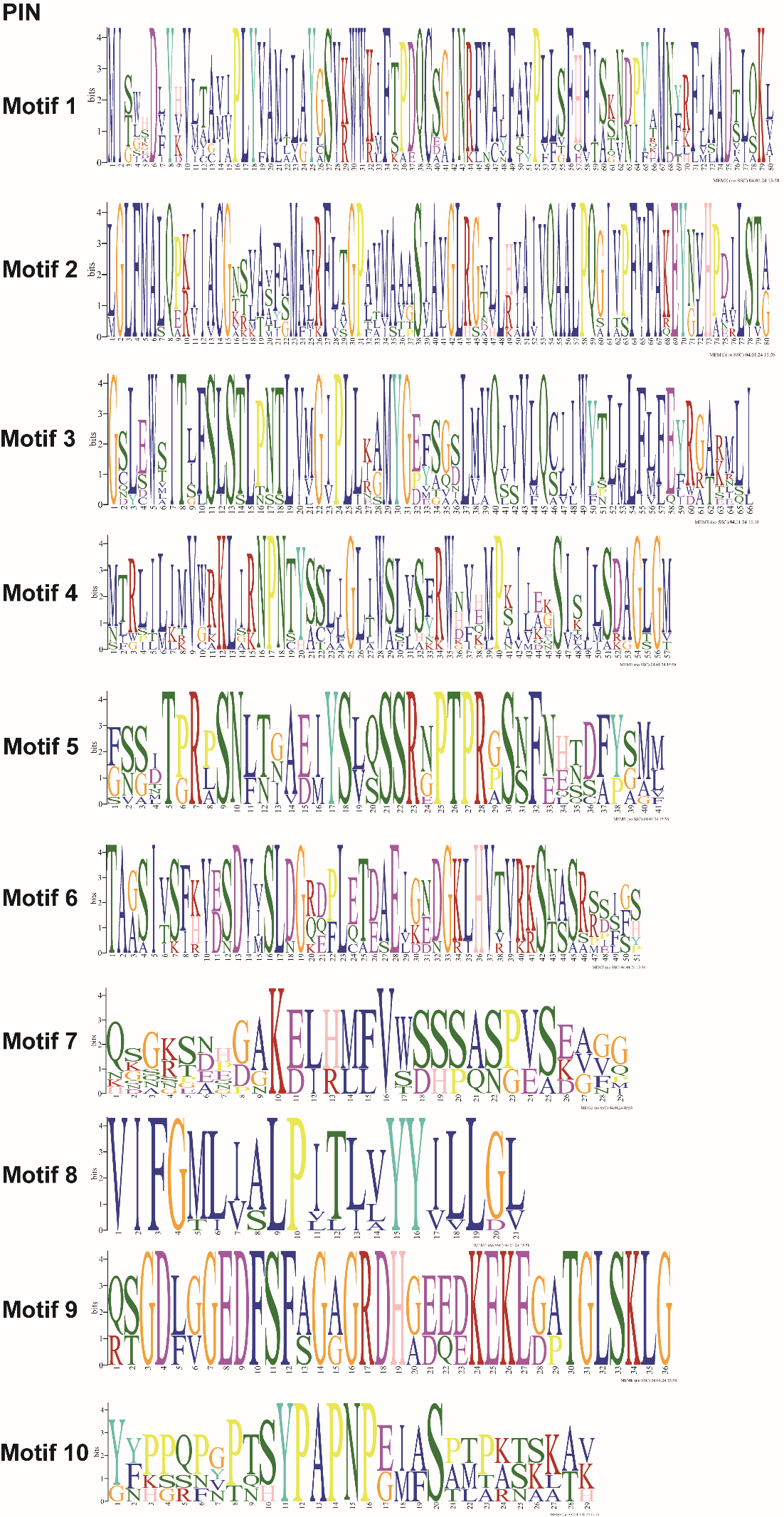


**Figure S8.** Ten main conserved motifs of SiPIN proteins.


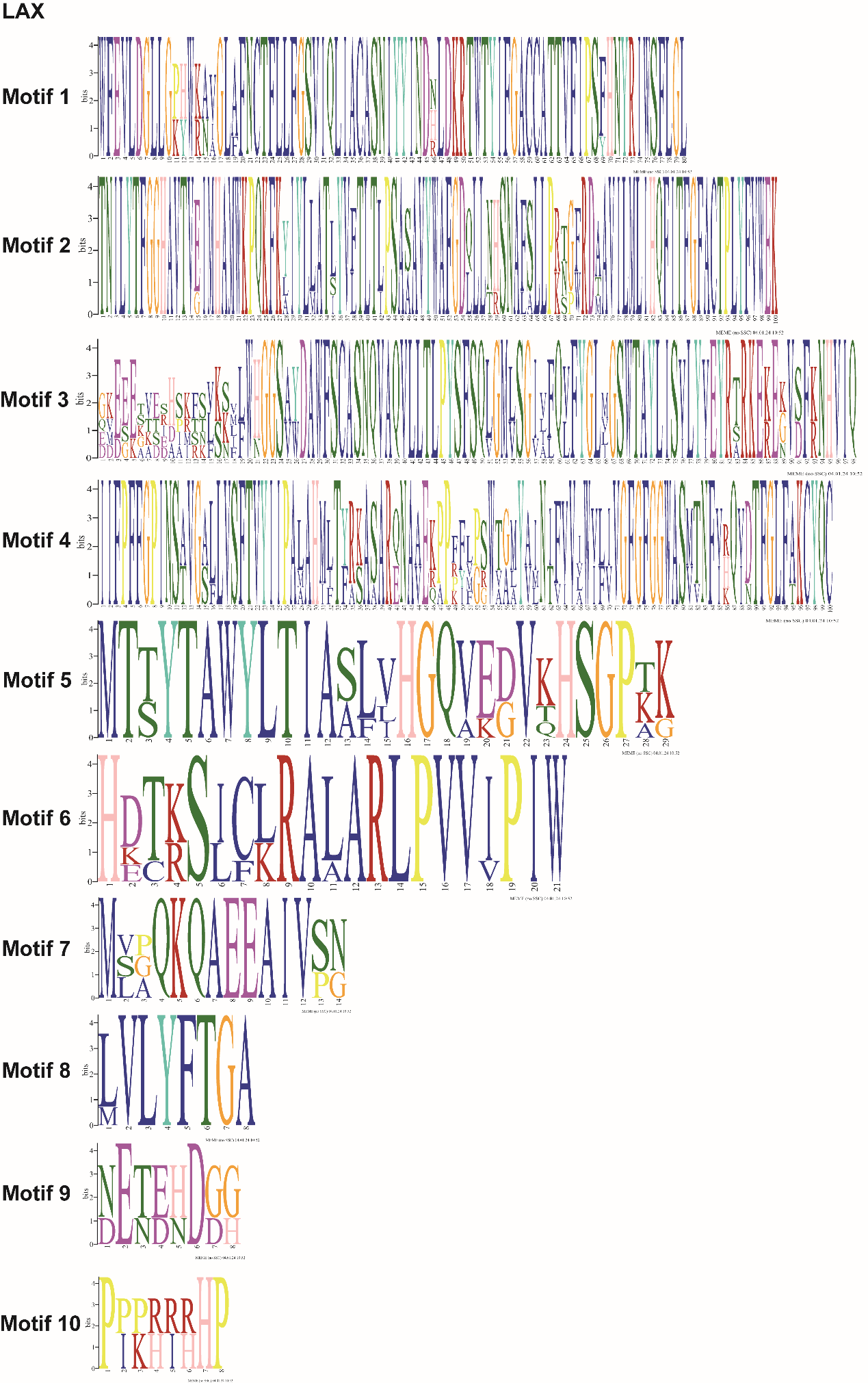


**Figure S9.** Ten main conserved motifs of SiLAX proteins.


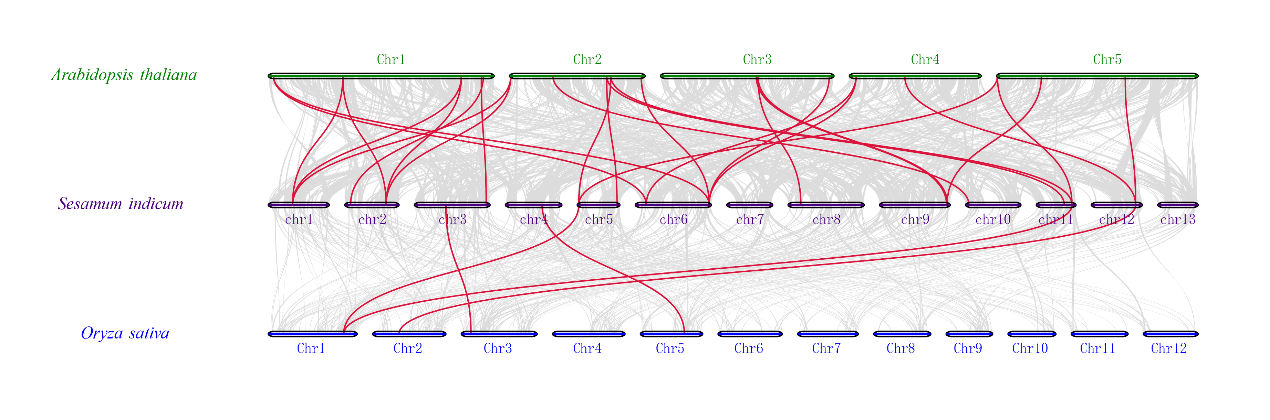


**Figure S10.** Collinearity analysis of *SiABCB/SiPIN/SiLAX* genes between *A. thaliana*, *S. indium* and *O. sativa*. Gray lines in the background represent the collinear blocks within the genomes of *A. thaliana*, *S. indium* and *O. sativa*, while the red lines show the collinear *SiABCB/SiPIN/SiLAX* gene pairs.


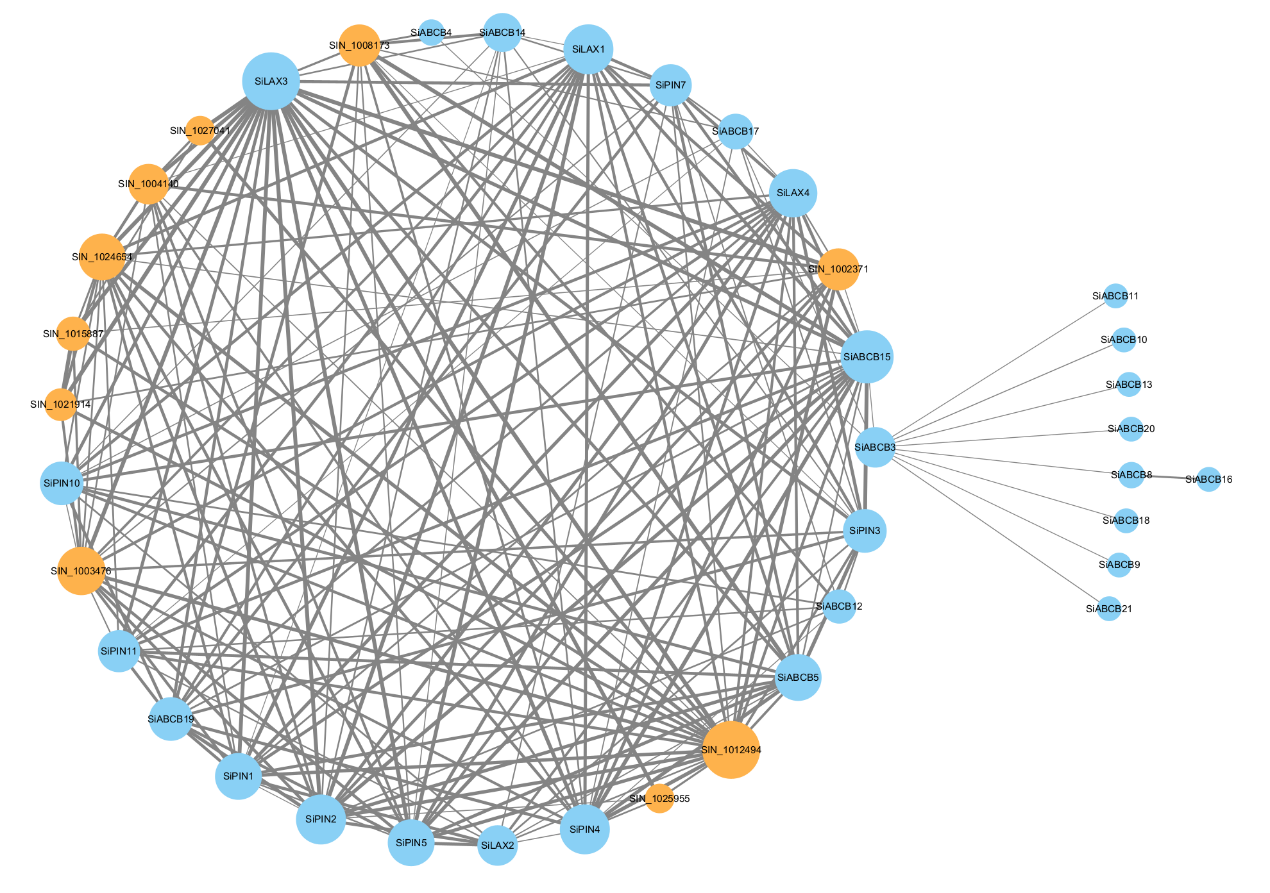


**Figure S11.** The interaction network of SiABCB/SiPIN/SiLAX protein in *S.indicum*. The blue dots are SiABCB/SiPIN/SiLAX protein. The yellow dots are other proteins interacting with SiABCB/SiPIN/SiLAX. The size of the dots represents the degree of interaction, and the thickness of the lines represents the strength of protein-protein interactions.


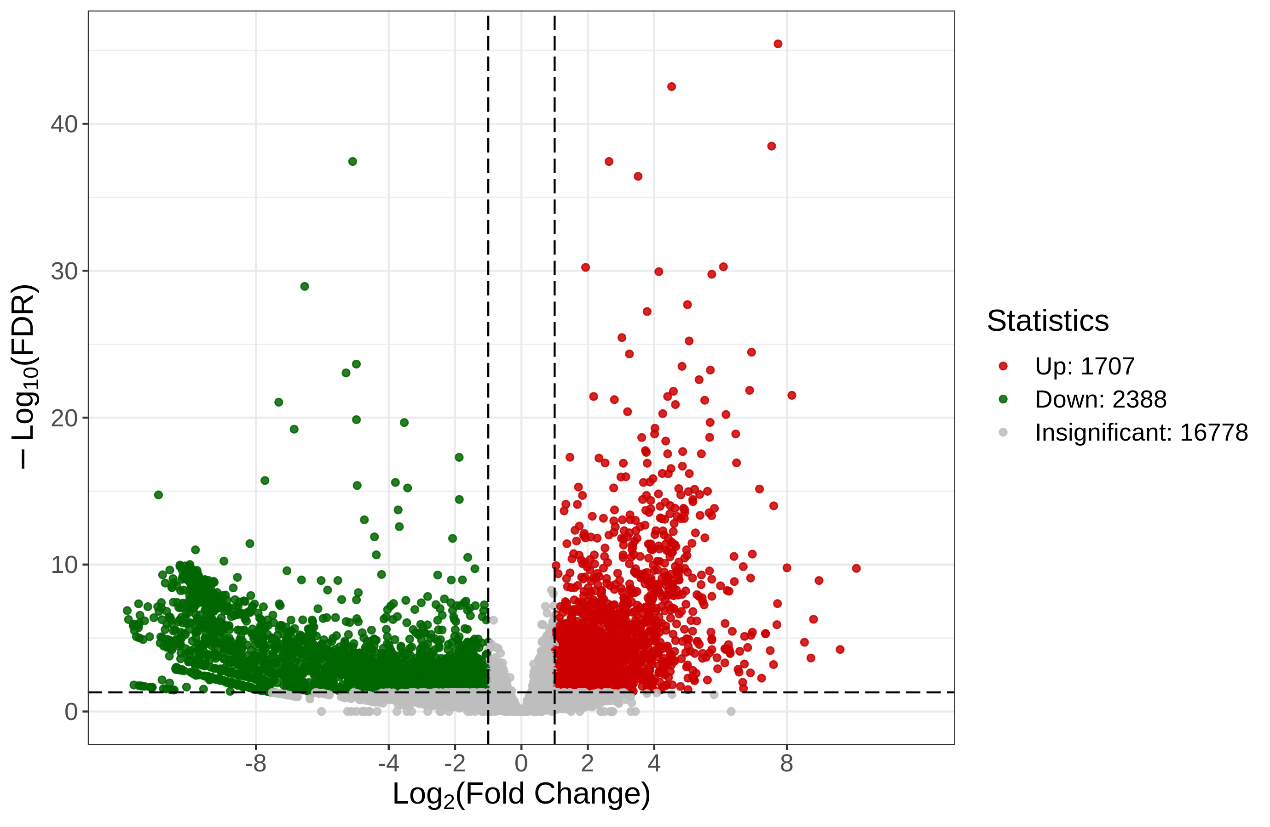


**Figure S12.** Volcano plot of differential genes in G1358 and LZ1 leaves. The horizontal axis represents the fold change in gene expression, and the vertical axis represents the significance level of differentially expressed genes. Red dots represent up-regulated differential genes, green dots represent down-regulated differential genes, and gray dots represent non-differentially expressed genes.


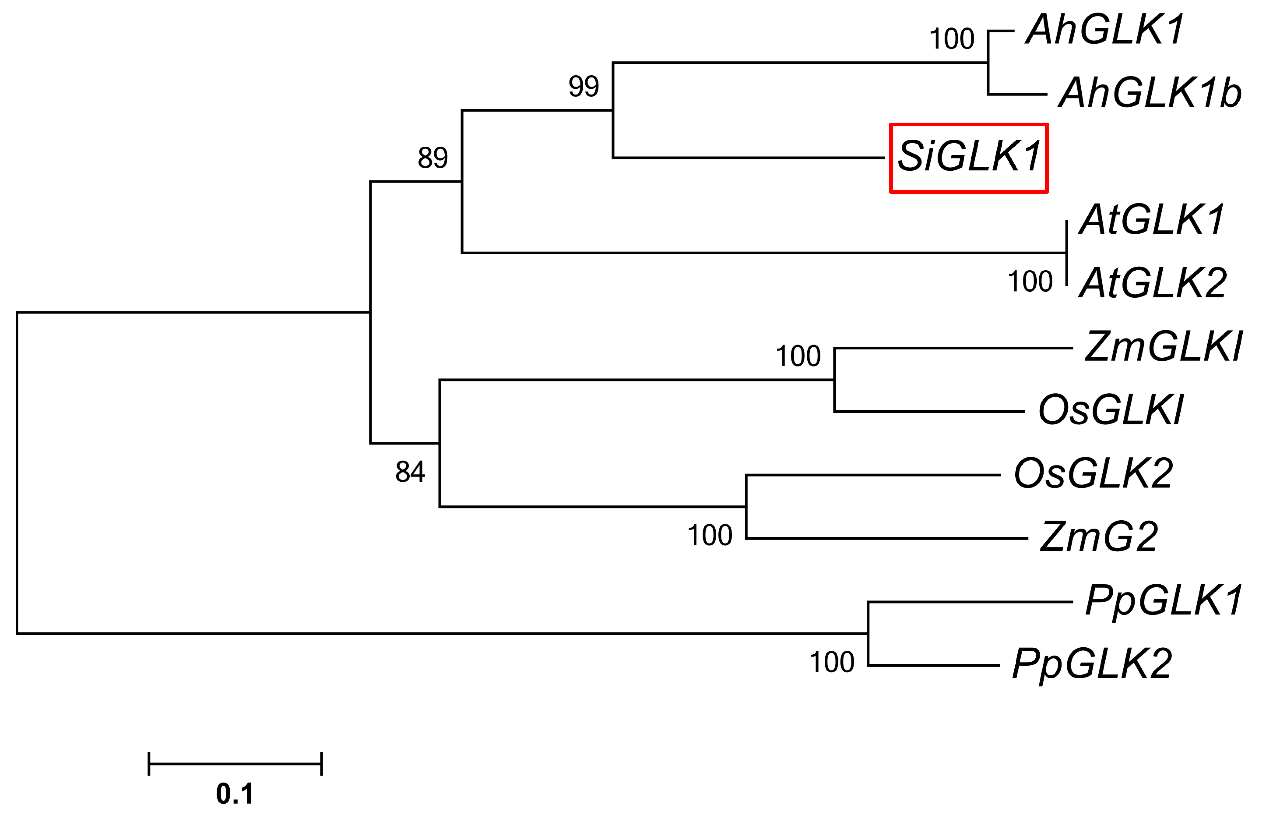


**Figure S13.** Phylogenetic tree of GLKs. Multiple sequence alignment of GLKs was performed using ClustalW. MEGA11 was used to construct the Neighbor-Joining (NJ) tree with 1000 bootstrap replicates. The red box represents the GLK of *S. indicum*. Ah: *Arachis hypogaea*; Si: *S. indicum*; At: A*rabidopsis thaliana*; Zm: *Zea mays*; Os: *Oryza sativa Japonica*; Pp: *Physcomitrella Patens*.


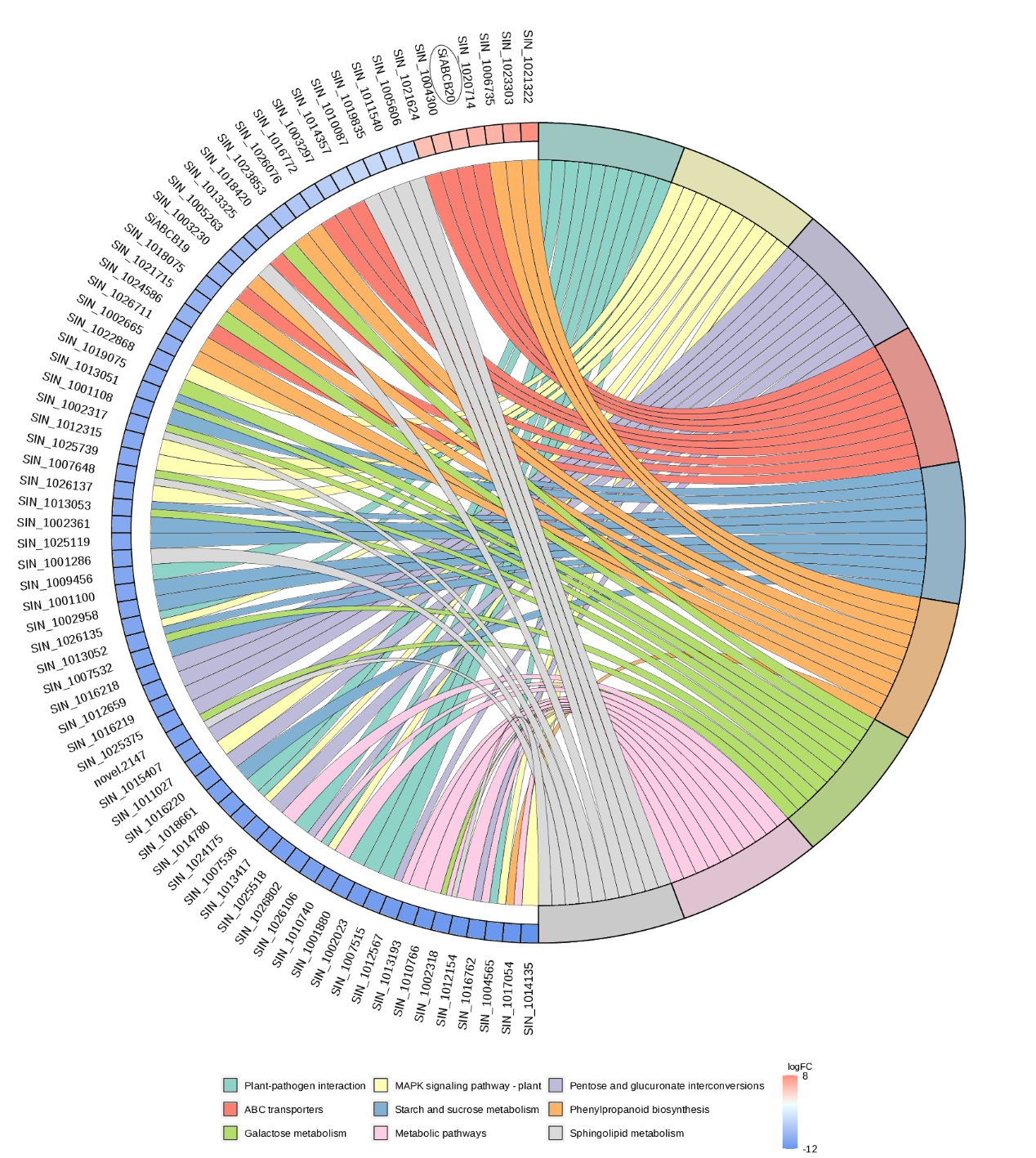


**Figure S14.** KEGG enrichment analysis of DEGs in G1358 and LZ1. The left side shows the top 10 genes of |log_2_FC| in each category. The right side shows the most significantly enriched 9 pathways. The middle line represents the corresponding relationship between pathways and genes. The legend of the heat map shows the log_2_FC value of genes, red represents the up-regulated gene, blue represents the down-regulated gene, and the color depth indicates the size of log_2_FC. The darker the color, the greater the difference multiple.
